# Development of Insulin and Leptin Resistance in the Mouse Brainstem with Age

**DOI:** 10.1007/s12035-025-05392-5

**Published:** 2026-01-16

**Authors:** Elvira De Frutos González, Nuria Lauzurica, José Joaquín Ochoa Navarro, Miriam García San Frutos, Fernando Aguado, Teresa Fernández-Agulló

**Affiliations:** 1 Area of Physiology, Faculty Health Sciences, Rey Juan Carlos University, Alcorcón, Madrid Spain; 2 Department of Cell Biology, Physiology and Immunology, Faculty of Biology, University of Barcelona, Barcelona, Spain; 3 Institute of Neurosciences, University of Barcelona, Barcelona, Spain

**Keywords:** Brainstem, Insulin resistance, Leptin resistance, Age, Neuroinflammation

## Abstract

Physiological aging involves a progressive deterioration of homeostatic mechanisms that cause obesity and defective glucose homeostasis, which develop age-related diseases increasing mortality risk and reducing lifespan. The brainstem is involved in glucose and metabolic homeostasis by integrating peripheral signals such as insulin and leptin. Here, we evaluated the brainstem response to intracerebroventricular administration of insulin or leptin and the relationship with physiological levels of key molecules implicated in their signal transduction pathway and inflammation in 3-, 6-, and 12-month-old mice which progressively increase adiposity and develop signs of insulin resistance. The initial steps of insulin and leptin signaling pathways decline with age, as well as the protein kinase B (Akt) phosphorylation response. Both hormones decrease the phosphorylation of AMP-activated protein kinase (AMPK) but, while the response to insulin increases with age, the response to leptin decreases in older animals. This insulin and leptin resistance is accompanied by changes in basal protein expression or phosphorylation of insulin and leptin receptors and insulin receptor substrates-1 (IRS-1), as well as the imbalance between basal levels of Akt-phosphorylated and non-phosphorylated protein, without changes in other serine kinases and/or inflammatory pathways such as glycogen-synthase-kinase-3 (GSK3), mammalian targets of rapamycin (mTOR), kinase-p70S6 (p70), protein kinase-C-ε (PKCε), p38 mitogen-activated protein kinase (p38), or c-Janus N-terminal kinase (JNK). High levels of proinflammatory cytokines and glial cell activation suggest the development of neuroinflammation in the brainstem with age, which could mediate the age-associated insulin and leptin resistance and the impairment in glucose and metabolic homeostasis commonly observed in the aging process.

**Graphical Abstract:**

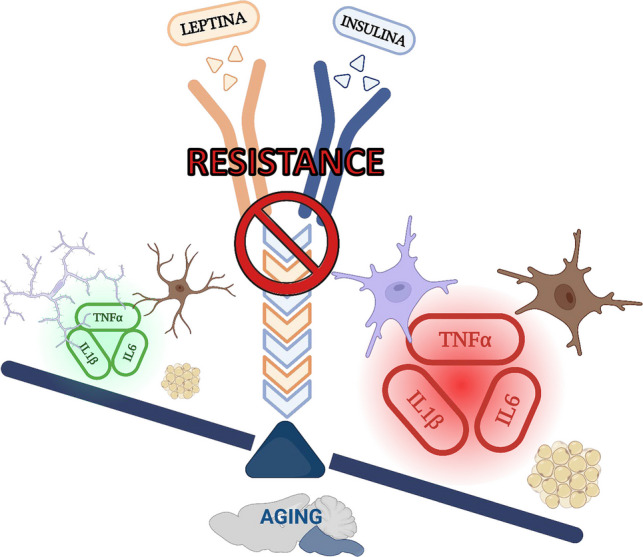

**Supplementary Information:**

The online version contains supplementary material available at 10.1007/s12035-025-05392-5.

## Introduction

Aging is a complex biological process characterized by progressive physiological changes that, in the context of the central nervous system (CNS), are associated with alterations in cellular metabolism, neurotransmitter systems, and neuronal plasticity which can lead to cognitive decline, motor impairments, and disruptions in homeostatic regulation [1].

As organisms age, resistance to both insulin and leptin often develops, characterized by a reduced responsiveness of target tissues to these hormones, which leads to age-related obesity and various pathologies [2]. Insulin and leptin are classically regarded as peptide hormones involved in metabolic regulation and energy homeostasis [3]. The two hormones have been described as adiposity signals [4], with complex functions not only in the periphery, but also at the level of the CNS where they regulate energy homeostasis and various other processes [5, 6]. Plasmatic insulin levels tend to better correlate with visceral adipose tissue, but leptin secretion reflects total fat mass, especially subcutaneous fat.

Insulin, traditionally recognized for its role in glucose metabolism, also has important functions in the CNS, including neuroprotection and cognitive processes [2, 7]. On the other hand, insulin is known to play a critical role in regulating peripheral glucose and metabolic homeostasis through activation of its receptors expressed in the hypothalamus [8–10].

Leptin, produced by adipose tissue, is involved in a negative feedback loop that suppresses food intake [11], decreases feeding behavior, increases thermogenesis [10], and plays a role in the regulation of body weight and composition, acting also in the hypothalamus [10, 12]. In addition, leptin and insulin conduct their actions through extrahypothalamic regions of the brain, including the brainstem [3, 13–21].

The brainstem, a critical region of the CNS, is responsible for controlling essential autonomic functions such as respiration, heart rate, blood pressure, and arousal [22]. Its involvement in metabolic regulation is particularly significant, as it integrates signals from peripheral organs and various brain centers to modulate energy balance and glucose homeostasis [23, 24].

In relation to this, the dorsal vagal complex (DVC), located in the dorsal medulla, comprises three structures: the nucleus of the solitary tract (NTS), the area postrema (AP), and the dorsal motor nucleus of the vagus nerve (DMV). The DVC is a gateway for many primary afferents from cardiovascular, respiratory, gastrointestinal, and other visceral sensory receptors [25, 26]. In turn, the vagus nerve innervates internal organs including the heart, lungs, gastrointestinal tract, and the NTS via the nerve bundle called the solitary tract. NTS neurons project locally to preganglionic motor neurons in the DMV and to other sites in the brainstem and hypothalamus to give a physiological response to incoming signals [27]. For all of this, the NTS is pointed out as the origin for CNS processing of cardiovascular, respiratory, glucoregulatory, and satiety signaling [28–31].

Additionally, within the brainstem oral and viscerosensory information ascends from the NTS [32, 33] to the parabraquial nucleus (PBN), which, in turn, sends and receives projections from several hypothalamic nuclei implicated in metabolic homeostasis [32, 34]. Besides, the dorsal raphe nucleus (DRN), the primary source of central serotonin 5-hydroxytryptamin [35], regulates many behavioral and physiological processes involved in energy homeostasis, primarily by promoting satiety to regulate appetite [36].

Both insulin receptor (IR) and leptin receptor (LEPR) have been located in the brainstem [13, 37]. Insulin signaling in the DMV regulates hepatic glucose production and food intake [38]. Leptin action on the NTS increases satiety due to reciprocal connections with hypothalamic nuclei (arcuate nucleus, dorsomedial hypothalamic nucleus, and paraventricular hypothalamic nucleus) that control food intake. On the DRN, leptin has a similar effect by increasing serotonergic activity. While on the PBN, it also contributes to glycemic control via projections to the ventromedial nucleus of the hypothalamus [39]. Additionally, leptin signaling in several hindbrain nuclei has been implicated in the regulation of thermogenesis and overall thermoregulatory processes [40].

Insulin and leptin exert their effects through complex signaling pathways [41]. Once insulin binds to its receptor, it regulates various cellular processes, including glucose uptake, glycogen synthesis and protein synthesis through insulin receptor substrates (IRS), phosphatidylinositol 3-kinase (PI3K), and protein kinase B (Akt) [7]. On the other side, the mitogen-activated protein kinases (MAPK) pathway including extracellular signal-regulated protein kinase 1/2 (ERK1/2) influences cell growth, survival, and expression of genes associated with glucose metabolism [42, 43]. Leptin signaling is mediated through the Janus kinase 2/signal transducer and activator of transcription 3 (JAK2-STAT3) pathway, as well as the IRSs, PI3K/Akt, and MAPK pathways [3]. In particular, the phosphorylation of STAT3 is necessary for regulating energy homeostasis [44]. Insulin and leptin signaling cascades overlap at the level of PI3K [8] to regulate glucose metabolism [45]. By this pathway, both inhibit the AMP-activated protein kinase (AMPK), essential for the anorexigenic and weight loss effects of insulin and leptin in the hypothalamus [46, 47].

Impairment of their function can occur in the presence of insulin and leptin resistance, both peripherally and/or centrally. Central insulin and leptin resistance could involve alterations in their transport across the blood–brain barrier and/or the blood cerebrospinal fluid barrier, as well as in their signaling pathways. In this sense, an increase in protein tyrosine phosphatases or serine/threonine kinases (PKA, mammalian targets of rapamycin (mTOR), its downstream kinase p70S6 (p70), ERK1/2, and some members of the protein kinase C (PKC)) has been implicated [48, 49]. In the case of LEPR, the retro-inhibitors of the JAK2–STAT3 pathway, the suppressor of cytokine signaling 3 (SOCS3), are systematically upregulated along with leptin resistance [50].

In addition to obesity and insulin resistance, chronic low-grade inflammation is another hallmark of the aging process termed “inflammaging” [51, 52]. This state of increased production of proinflammatory cytokines and the activation of inflammatory signaling pathways leads to serine phosphorylation of IR and IRSs through N-terminal kinase (JNK), p38 MAPK (p38), and the inhibitory kappa β kinase (IKKβ) [45]. Moreover, inflammatory signals acting through the IKKβ-NFkB pathway may also induce insulin resistance by transcriptional mechanisms [53, 54].

Epidemiologic studies have identified abdominal obesity as a major risk factor for insulin resistance which triggers age-related diseases such as diabetes, cardiovascular diseases, Alzheimer’s disease, and some cancers, increasing mortality risk and reducing lifespan [55]. Furthermore, structural changes in the brain associated with the accumulation of visceral fat have been attributed to inflammation [56].

Glial cells, particularly astrocytes and microglia, play crucial roles in maintaining brain health and function.

Several studies have reported, using immunohistochemistry, changes in glial fibrillary acidic protein (GFAP) expression, a marker of astrocytes, in the NTS, induced by different experimental stimuli and across a wide range of disease models associated with inflammation [57]. Furthermore, blockade of microglial activation also affects GFAP changes, indicating crosstalk between these two glial cell types in the NTS [58]. Age-related increases in GFAP immunoreactivity within the NTS have been documented in rats, suggesting a potential link between astrocytic activation and physiological alterations associated with aging [59]. Both cell types potentially contribute to the altered inflammatory state and metabolic dysregulation observed in the aging brain [60, 61].

Finally, and as previously indicated, insulin and leptin exert distinct physiological effects that are highly dependent on the specific cell types and neural circuits they target. Most of the data regarding aging-induced obesity and insulin and leptin resistance have been described in areas related to ingestive behavior, like hedonic centers or the hypothalamus. However, less is known about the alterations at the level of the brainstem. In this study, we aim to investigate age-related changes in insulin and leptin signaling, their potential association with inflammation, and the relationship with glial cells in the brainstem of C57BL6 mice from 3, 6, and 12 months of age. As the population continues to age globally, understanding the molecular and cellular mechanisms underlying the brainstem in early aging becomes increasingly important for the essential functions it performs. This knowledge could pave the way for developing targeted interventions to maintain brainstem function and prevent age-related disorders.

## Materials and Methods

### Animals

Three-, 6-, and 12-month-old male C57BL6 mice fed standard laboratory chow and water, both available ad libitum, were used throughout this study. Mice were housed in climate-controlled quarters with a 12-h light, 12-h darkness cycle. All animal experimentation was conducted in accordance with the Spanish guidelines for care and use of laboratory animals and protocols approved by the URJC and Community of Madrid (PROEX 068/16; PROEX 001.8/23).

### Adiposity and Metabolic/Biochemical Assessments

White adipose tissues (WATs), including epididymal (eWAT), perirenal (pWAT), and inguinal subcutaneous (sWAT), as well as brown adipose tissue (BAT), were collected and weighed from all experimental animals sacrificed without prior fasting. eWAT was carefully dissected from the ventral region adjacent to the testes, separated from surrounding connective tissues, and weighed immediately. pWAT was isolated from the area surrounding the kidneys and weighed. sWAT was collected from the inguinal region beneath the skin along the lower abdominal area, dissected, cleaned of non-adipose components, and weighed. BAT was extracted from the interscapular region located between the scapulae, ensuring complete removal from underlying muscle and connective tissues, and weighed. The visceral adiposity (%) was calculated by summing the eWAT and pWAT in relation to the body weight of each animal. Additionally, the longitudinal measurement of each animal was recorded to assess its body length.

Blood samples were collected from all experimental animals, anesthetized, and fasted overnight. Blood glucose was measured immediately before sacrifice via the tail vein of the mice using a glucose analyzer (Contour next, Bayer). Serum insulin and leptin levels were quantified using commercial ELISA kits (Millipore EZMI-13 K; EZML-82 K; respectively) according to the manufacturer’s instructions.

### Metabolic Tolerance Test (MTTs)

#### Glucose Tolerance Test (GTT)

Glucose solution was prepared for intraperitoneal (i.p.) injection at a dose of 1 mg/g body weight, with the injection volume not exceeding 200 µL. The injection was performed after fasting overnight. A blood glucose analyzer was used to measure glucose levels from the tail vein as previously described. Baseline blood glucose was recorded immediately before glucose administration (0 min), followed by measurements at 10, 20, 30, and 60 min post-injection.

#### Insulin Tolerance Test (ITT)

Insulin solution was prepared for i.p. injection at a dose of 0.75 mU/g body weight, with the injection volume not exceeding 200 µL. The injection was performed after fasting overnight. A blood glucose analyzer was used to measure glucose levels from the tail vein. Baseline blood glucose was recorded immediately before insulin administration (0 min), followed by measurements at 10, 20, 30, and 60 min post-injection.

#### Pyruvate Tolerance Test (PTT)

Pyruvate solution was prepared for i.p. injection at a dose of 0.75 mU/g body weight (2 g/kg), with the injection volume not exceeding 200 µL. The injection was performed after fasting overnight. A blood glucose analyzer was used to measure glucose levels from the tail vein. Baseline blood glucose was recorded immediately before insulin administration (0 min), followed by measurements at 10, 20, 30, and 60 min post-injection.

#### HOMA-IR

The homeostasis model assessment of insulin resistance index (HOMA-IR) was calculated using fasting insulin and glucose levels. The formula applied was as follows: HOMA-IR = fasting insulin (µIU/mL) × fasting glucose [(mmol/L)/22.5]. The calculation and interpretation of HOMA-IR were performed according to established protocols and previously described [62, 63].

### Intracerebroventricular (i.c.v.) Administration of Insulin and Leptin

After fasting overnight, animals were first anesthetized with an i.p. injection of pentobarbital sodium (Dolethal, 0.065 g/kg). When corneal and patellar reflexes were completely lost, they were placed into an animal stereotaxic frame. An incision of the scalp was made in order to expose the skull, and a dental drill was used to make an opening for the insertion of a Hamilton needle (10-µl syringe) into the right lateral ventricle of the brain (0.4 mm posterior to the bregma, 1.0 mm lateral to the midline, 2.4 mm below the surface of the skull). Based on protocols adapted from the study by Mizoguchi et al. [64], leptin (1 µg/µl), insulin (5.97 µM), or saline was administered in a volume of 2 µl over 1 min; animals were decapitated after 30 min from the beginning of the infusion and the brainstems were dissected, frozen in liquid nitrogen, and stored at −80 °C until use.

### Immunohistochemistry

Mice were anesthetized with Dolethal, as previously described in i.c.v. methods. Transcardial perfusion was performed with a fixative solution of 4% paraformaldehyde in phosphate buffer pH 7.4. Brains were post-fixed for 4 to 6 h in the same fixative solution and then transferred to a 30% sucrose solution overnight. The brains were then frozen using Tissue-Tec OCT (Sakura). Coronal section 25 µm spaced were collected along the whole brainstem (between −3.88 and −8 mm bregma according to Paxinos) as eight 1-in-4 series, placing them in tissue culture wells filled with cryoprotectant (50% phosphate buffer, 30% ethylene glycol, 20% glycerol; pH 7.4) using a sliding microtome with fast freezing unit (Microm HM 400 R) and stored at −20 °C until further processing. Two series per animal were processed for different antibodies.

Free floating sections were blocked with 0.3% Triton X-100 (30 min) and with 1:100 gelatine (1 h) at room temperature and incubated overnight at 4 °C with anti-Ob-R (1:1000) for LEPR, anti-GFAP (1:7500) for astrocytes, or anti-ionized calcium binding adaptor molecule 1 (Iba1) (1:1000) for microglia detection (Table 1). The next day, sections were incubated with anti-goat (1:500, Vector Laboratories) and anti-rabbit (1:500, Vector Laboratories) for 1 h at room temperature. After being washed in phosphate buffer saline (PBS), sections were processed according to the immunoperoxidase procedure (VECTASTAIN Elite ABC system; Vector Laboratories) using the glucose oxidase-DAB-nickel method [65]. Elapsed times for processing all sections from the different mice were the same.

**Table 1:** Antibodies used for immunostaining and Western blotting

Antibody	Host	Source	Catalog number
Akt1/2/3 (5C10)	Rabbit polyclonal	Santa Cruz Biotechnology	sc-8312
AMPKα	Rabbit polyclonal	Santa Cruz Biotechnology	sc-2532
ERK1/2	Rabbit polyclonal	Sigma-Aldrich®	06–182
GFAP	Rabbit polyclonal	DAKO	Z0334
GSK-3β	Mouse monoclonal	BD Transduction Laboratories™	610201
Iba1	Rabbit polyclonal	Genetex	GTX100042
Insulin Rβ (C-19)	Rabbit polyclonal	Santa Cruz Biotechnology	sc-711
IRS-1 (C-20)	Rabbit polyclonal	Santa Cruz Biotechnology	sc-559
IRS-2 (H-205)	Rabbit polyclonal	Santa Cruz Biotechnology	sc-8299
JNK2 (56G8)	Rabbit monoclonal	Cell Signaling Technology®	#9258
Leptin receptor (LEPR)	Rabbit polyclonal	Abcam	Ab5593
mTOR	Rabbit polyclonal	Cell Signaling Technology®	#2972
NFκβ p65 (C-20)	Rabbit polyclonal	Santa Cruz Biotechnology	sc-372
Ob-R (M-18)	Goat polyclonal	Santa Cruz Biotechnology	Sc-1834
p38 MAPK	Rabbit polyclonal	Cell Signaling Technology®	#9212
p70 S6 Kinase	Rabbit polyclonal	Cell Signaling Technology®	#9202
Phospho-Akt1/2/3 (Ser 473)-R	Rabbit monoclonal	Cell Signaling Technology®	#4058S
Phospho-AMPKα (Thr172)	Rabbit polyclonal	Cell Signaling Technology®	#2531
Phospho ERK1/2	Rabbit monoclonal	Sigma-Aldrich®	05-797R
Phospho-GSK-3β (Ser9)	Rabbit polyclonal	Cell Signaling Technology®	#9336
Phospho-Insulin Rβ (Tyr 1162/1163)	Rabbit polyclonal	Santa Cruz Biotechnology	sc-25103-R
Phospho-mTOR (Ser2448)	Rabbit polyclonal	Cell Signaling Technology®	#2971
Phospho-PKCε (Ser 729)	Rabbit polyclonal	Santa Cruz Biotechnology	sc-12355-R
Phospho-p38 MAPK (Thr180/Tyr182)	Rabbit polyclonal	Cell Signaling Technology®	#9211
Phospho-p70 S6 Kinase (Thr389)	Rabbit polyclonal	Cell Signaling Technology®	#9205
Phospho-SAPK/JNK (Thr183/Tyr185)	Rabbit polyclonal	Cell Signaling Technology®	#9251
Phospho-STAT3 (Tyr705) (D3A7)	Rabbit monoclonal	Cell Signaling Technology®	#9145
PKCε (C-15)	Rabbit polyclonal	Santa Cruz Biotechnology	sc-214
PTEN (A2B1)	Mouse monoclonal	Santa Cruz Biotechnology	sc-7974
SOCS3	Mouse monoclonal	Santa Cruz Biotechnology	sc-51699
STAT3 (126H6)	Mouse monoclonal	Cell Signaling Technology®	#9139
β-Actin Clone AC-15	Mouse monoclonal	Sigma-Aldrich®	A5441

After several washes in PBS, sections were mounted in slide glasses, dried, dehydrated gradually in ethanol, cleared in xylene, and covered with Eukitt (Sigma-Aldrich). In control sections, where the primary antibody was not used, no immunoreaction was observed.

Regions of interest (ROIs) for each animal were photographed, using a Zeiss Axioplan 2 microscope with a Leica adapted camera (type DFC7000 T), from the left and right sides of the brainstem and included DRN, PBN, and DVC (AP, NTS and DMV). ROIs were acquired on a light, at 5×, 20×, 40×, and 100 × magnification and saved as 24-bit TIFF images. Care was taken to ensure that all photomicrographs were captured under similar illumination conditions. For more detailed images in LEPR, Iba1, and GFAP immunostaining, 100 × and 40 × magnification were used, and Z-stack images were acquired by sequentially focusing from the top to the bottom of each section (between 4 and 8 pictures were acquired). The resulting images were then combined into a single stack using ImageJ software (National Institute of Health (NIH)), allowing for the integration of all focal planes into one comprehensive image to ensure that all layers were visualized simultaneously.

In all cases, morphological evaluations of LEPR, Iba1, and GFAP immunoreactivity on DAB-stained brainstem slices in each ROI were performed by two independent observers in a blinded manner, using 3-month-old animals as the reference point. All images at 20 × magnification for the same nucleus (three slices for each nucleus of both sides) were analyzed per animal (*n* = 3–4 for LEPR and 2–3 for Iba1 and GFAP) using identical acquisition microscope parameters. The intensity of the immunostaining was subjectively scored as lower (−), much lower (− −), or higher (+), or much higher (+ +) compared with 3-month-old animals. Changes were only recorded when there was agreement between the two observers. Additionally, we conducted a semiquantitative analysis by measuring the positive percentage area within each ROI using ImageJ® software (NIH, ML, USA), and data were represented as % vs. 3-month-old group.

### Western Blot Analysis

Untreated animals for the study of basal protein expression and phosphorylation were anesthetized using i.p. injection of ketamine (112.5 mg/kg)/xylazine (4.5 mg/kg). Once there was no patellar or corneal reflex, animals were decapitated, the brain was dissected, and the brainstem was frozen in liquid nitrogen and stored at −80 °C. Brainstems from basal or i.c.v.-treated animals were homogenized in lysis buffer (1 mM EDTA, 1 mM EGTA, 50 mM TRIS, 10 mM β-glycerol, 50 mM NaF, 5 mM pyrophosphate Na, 0.1% βMetOH, and 1% Triton X-100) with phosphatase and protease inhibition cocktail (0.1 mM PMSP, 1 µg/ml Aprotinin, 1 µg/ml benzamidine, 1 µg/ml leupeptin, 0.1 µM okadaic acid, and 0.5 mM Na_3_VO_4_), incubated for 30 min with shaking at 4 °C, and centrifuged (20,000 g/30 min 4 °C). Resultant protein suspensions were measured using Coomassie Protein Assay (Thermo Fisher Scientific). Twenty to sixty micrograms of protein were separated by weight using electrophoresis SDS-PAGE gel and transferred to a PVDF membrane (Immune-blot, Bio-Rad) in transferred buffer (25 mM Tris, 120 mM glycine and 20% MeOH). Membranes were blocked with 5% of BSA (Sigma-Aldrich) or in non-fat dry milk in tween-tris-buffered saline (TTBS; 0.1% Tween 20, 50 mM Tris–HCl, 150 mM NaCl, pH 7.6). After blocking, membranes were incubated with the primary antibody (Table 1) overnight at 4 °C, washed three times, and incubated for 2 h at room temperature with HRP-conjugated anti-rabbit, anti-mouse, or anti-goat IgG secondary antibodies (1:5000; Sigma-Aldrich) all in TTBS. Proteins of interest were immunodetected by chemiluminescence using clarity Western ECL substrate (Bio-Rad) and ChemiDoc Imaging system (Bio-Rad). Protein expression was quantified using ImageJ software (NIH).

Once membranes were immunoblotted with phospho-IRβ, -STAT3, -Akt, -GSK3, -AMPK, -ERK1/2, -mTOR, -PKCε, -P38, -P70, and -JNK antibodies, they were stripped with Re-blot plus mild reagent (Millipore) for 15 min, washed three times for 10 min with TTBS, and then reblotted with the corresponding antibodies against total IRβ, STAT3, Akt, GSK3, AMPK, mTOR, PKCε, P38, P70, and JNK. In other cases, membranes were incubated with GFAP, Iba1, IRS-1, IRS-2, LEPR, NFκB p65, PTEN, and SOCS3 antibodies. For the quantification of protein expression, membranes were reblotted with anti-βActin antibody to normalize with respect to the total amount of protein. βActin levels do not vary with age when normalized to total membrane protein detected by Ponceau red staining.

To represent the data of i.c.v. experiments, protein phosphorylation levels were represented as % vs. the control group (saline). Additionally, the fold of response between saline and treated groups was calculated for each age group. Basal protein expression or phosphorylation was represented as % vs. the 3-month-old group.

### RT-qPCR

Brainstems were obtained and stored from animals anesthetized with ketamine/xylazine as previously described. Brainstems were homogenized in Tri Reagent (Sigma-Aldrich) followed by the addition of 1-bromo-3-chloropropane (100 µl/ml). After vigorous vortexing and 15-min incubation at room temperature, the samples were centrifuged at 14,000 rpm and 4 °C for 15 min. The aqueous phase was collected and mixed with isopropanol (500 µl/ml Tri Reagent) and then stored at −20 °C for 24 h. After centrifugation, the RNA pellet was washed with 75% ethanol in DEPC-treated water, vortexed, and centrifuged again. The RNA pellet was air-dried, resuspended in nuclease-free water, and stored at −80 °C until use.

RNA integrity and quantity were evaluated using a Nanodrop ND 1000 spectrophotometer and a RNA 6000 Nano Bioanalyzer. cDNA was synthesized from 2 ng of RNA using the High-Capacity cDNA Reverse Transcription Kit (Applied Biosystems). qRT-PCR was performed using Master Mix 1 × TaqMan or FastStart Universal SYBR-green reagents on a 7500 Real-Time PCR System (Applied Biosystems) along with specific TaqMan probes or primers listed in Tables 2 and 3, respectively. The PCR protocol included a 2-min step at 50 °C, 10 min at 95 °C, 40 cycles of 15 s at 95 °C, and 1 min at 60 °C. Reactions were conducted in triplicate, and results were normalized against the endogenous control S18.

**Table 2:** List of TaqMan probes used in RT-PCR studies

Probe name	Reference	Commercial supplier
LEPR	Mm00440181_m1	Fisher Scientific S.L

**Table 3:** List of primers used in RT-PCR studies

Probe name	Sequence 5′ → 3′	Commercial supplier
Aif1 (Iba1) Fdw	TGATCCCAAATACAGCAATGATGAG	Sigma-Aldrich
Aif1 (Iba1) Rev	TCCAGCATTCGCTTCAAGGAC	Sigma-Aldrich
GFAP Fdw	GATCGCCACCTACAGGAAAT	Sigma-Aldrich
GFAP Rev	GTTTCTCGGATCTGGAGGTT	Sigma-Aldrich
IL-1β (Interleukin 1 beta) Fdw	CGACAAAATACCTGTGGCCT	Sigma-Aldrich
IL-1β (Interleukin 1 beta) Rev	TTCTTTGGGTATTGCTTGGG	Sigma-Aldrich
IL-6 (Interleukin 6) Fdw	GTGGCTAAGGACCAAGACCA	Sigma-Aldrich
IL-6 (Interleukin 6) Rev	GGTTTGCCGAGTAGACCTCA	Sigma-Aldrich
TNFα Fdw	CATCTTCTCAAAACTCGAGTGACAA	Sigma-Aldrich
TNFα Rev	TGGGAGTAGATAAGGTACAGCCC	Sigma-Aldrich

### Statistics

Results are presented as the mean ± SEM (standard error of the mean). “n” refers to the number of animals per experimental group in each experiment. Statistical analyses were performed using the Student’s *t*-test, one-way or two-way ANOVA, and Dunnett’s multiple comparison test, considering differences significant when *p* < 0.05. GraphPad Prism 8 software was used for the analysis.

## Results

The characteristics of the 3-, 6-, and 12-month-old animals used in this study are summarized in Table 4. As can be seen, age is associated with an increase in body weight, length, and adiposity. All WAT pads studied significantly increase with age, including visceral adiposity. BAT tends to increase with age, but it does not reach statistical differences.

**Table 4:** Characteristics of the animals

	3-month-old	6-month-old	12-month-old
Body weight (g)	30.8 ± 0,66	35.17 ± 1.06^**^	36.93 ± 0.71^****^
Long (mm)	95.77 ± 0.83	100.57 ± 0.80^**^	102.78 ± 1.61^***^
sWAT (g)	0.50 ± 0.04	0.76 ± 0.10	0.99 ± 0.13^*^
eWAT (g)	0.85 ± 0.09	1.6 ± 0.19^*^	1.49 ± 0.16^*^
pWAT (g)	0.27 ± 0.03	0.49 ± 0.05^*^	0.51 ± 0.05^*^
Visceral adiposity (%)	3.75 ± 0.31	5.68 ± 0.43^**^	4.96 ± 0.25^*^
BAT (g)	0.11 ± 0.01	0.4 ± 0.04	0.16 ± 0.03
Serum leptin levels	1.29 ± 0.17	12.28 ± 5.93	36.17 ± 3.41^***^
Fasting blood glucose (mg/dl)	6.85 ± 0.51	7.54 ± 0.30	9.20 ± 0.36^***^
Serum insulin levels	1.15 ± 0.36	1,0.78 ± 0.09	3.28 ± 0.68^*^
HOMA-IR	0.32 ± 0.04	0.71 ± 0.21	2.08 ± 0.32^***^

The data represent means ± SEM of 4–7 animals per group. The effect of age between 3-, 6-, and 12-month-old mice was evaluated by one-way ANOVA test followed by Dunnett’s post hoc test (*p < 0.05, **p < 0.01, ***p < 0.001, ****p < 0.0001 vs. 3-month-old mice)

Adiposity is associated with elevated serum leptin levels. As is shown in Table 4, there is a progressive increase with age, much more evident in 12-month-old mice.

**Fig. 1:**
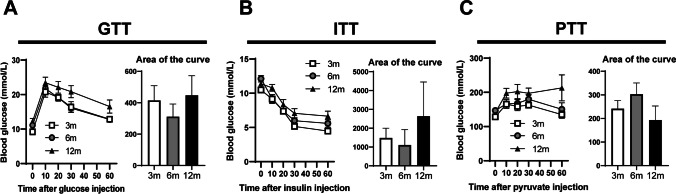
Metabolic tolerance test (MTTs). **A** Left, measurement of plasma glucose during glucose tolerance test (GTT); right, the calculated area of the curves. **B** Left, measurement of plasma glucose during insulin tolerance test (ITT); right, the calculated area of the curves. **C** Left, measurement of plasma glucose during pyruvate tolerance test (PTT); right, the calculated area of the curves. Left graphics show 3-month-old (filled rectangle), 6-month-old (filled circle), and 12-month-old (filled triangle). Differences across ages in the area of the curves were analyzed using one-way ANOVA followed by Dunnett’s post hoc test compared with 3-month-old mice. No significant differences were found. Data are presented as means ± SEM of 4–6 animals per group

Adiposity is also associated with some metabolic alterations in glucose homeostasis and insulin response; it is known that insulin resistance is one of the consequences of increased adiposity. In our model, fasting glucose levels increase with age, indicating that aged animals are not able to preserve glucose homeostasis. Moreover, the greater insulin levels observed in aged mice may indicate insulin resistance. When HOMA-IR was calculated, it showed a significant increase with age, suggesting that a state of insulin resistance is already present in 12-month-old animals. We further assessed glucose metabolism and insulin sensitivity by the administration of glucose (GTT), insulin (ITT), or pyruvate (PTT) (Fig. 1). In all cases, the response tends to worsen with age, although in no case were significant differences found (Fig. 1).

### Brainstem Insulin and Leptin Responses with Age

**Fig. 2:**
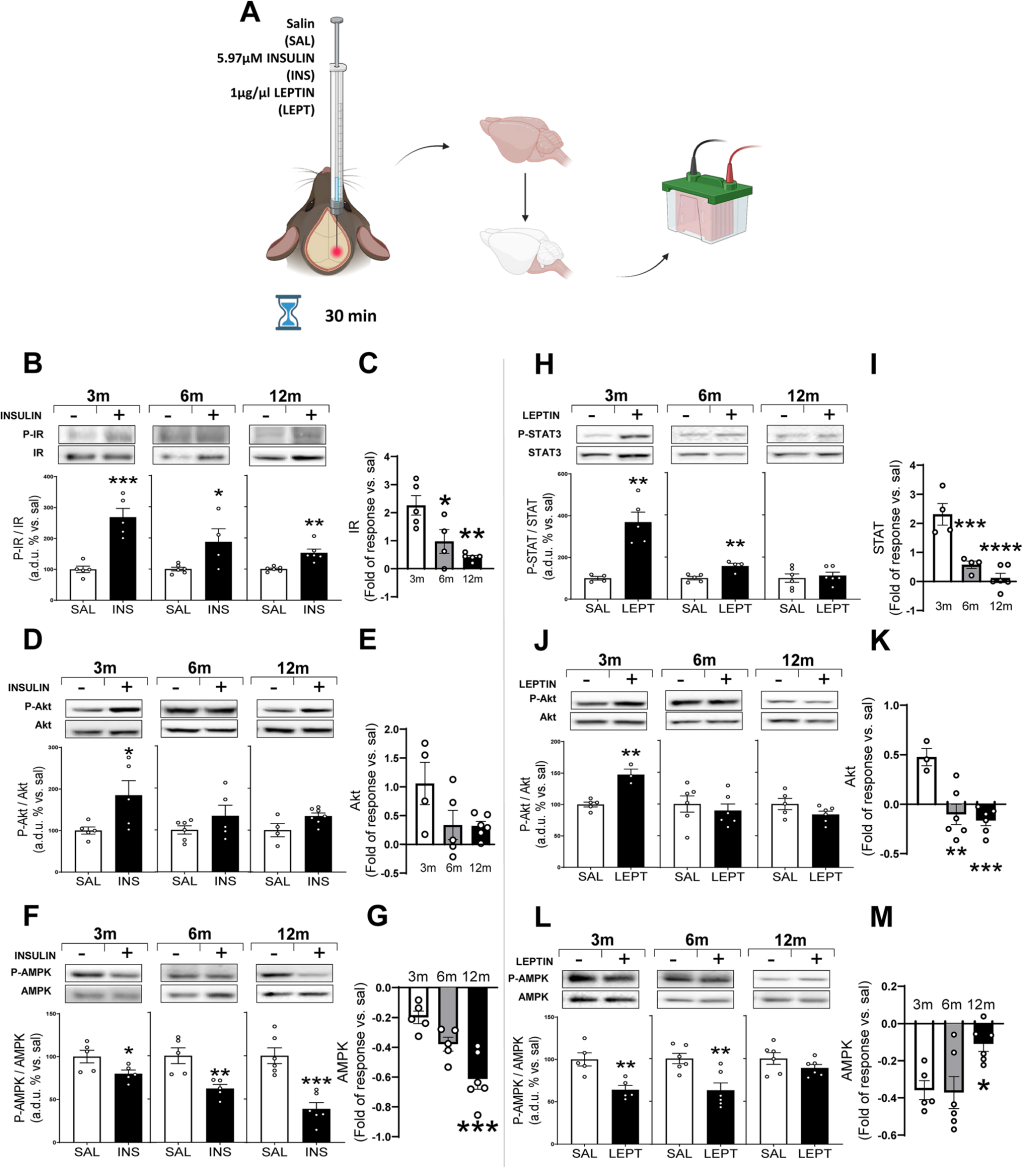
Brainstem insulin and leptin response. **A** Schematic representation of the intracerebroventricular (i.c.v.) injection procedure, followed by brainstem protein extraction and subsequent electrophoresis. **B**,** D**,** F** Densitometric evaluation of IR activation (P-IR/IR), AKT activation (P-AKT/AKT), and AMPK activation (P-AMPK/AMPK) proteins after 30 min of insulin i.c.v. injection; **H**,** J**,** L** Densitometric evaluation of STAT3 activation (P-STAT3/STAT3), AKT activation (P-AKT/AKT), and AMPK activation (P-AMPK/AMPK) after 30 min of leptin i.c.v. injection from whole brainstem extract of 3-, 6-, and 12-month-old mice. Results are expressed as arbitrary densitometric units (a.d.u.) relative to the control group (saline-injected animals), set to 100%. Insulin and leptin data were compared with age-matched controls using a non-paired Student’s *t*-test. *p < 0.05, **p < 0.01, ***p < 0.001 vs. saline group of the same age. **C**,** E, G**,** I, K**,** M** Fold of response to insulin or leptin treatment relative to saline control within each age group. Differences in fold of response across ages were analyzed using one-way ANOVA followed by Dunnett’s post hoc test. *p < 0.05, **p < 0.01, ***p < 0.001 compared with 3-month-old mice. Data are presented as means ± SEM of 4–6 animals per group

To elucidate if the age-related alteration in metabolism and glucose homeostasis could be related with insulin resistance in the brainstem with age, we administered insulin via i.c.v. injection and evaluated the phosphorylation of IR and Akt, major markers of insulin signaling, in mice of 3, 6, and 12 months of age after 30 min of treatment (Fig. 2A).

As we can see in Fig. 2B, insulin significantly increases IR phosphorylation in all ages compared with the control groups that received saline. Nevertheless, when we analyzed this increase as fold of response with respect to their saline (Fig. 2C), the ANOVA shows a significant effect of age, the IR response being significantly lower at 6- and 12- than in 3-month-old animals.

Insulin stimulation of Akt phosphorylation is shown in Fig. 2D. At 3 months of age, insulin significantly increases Akt phosphorylation whereas the stimulation does not reach statistical significance in 6- and 12-month-old animals. When the fold of response was compared by ANOVA, no significant differences with age are reached (Fig. 2E), probably due to the dispersion in the response of 3-month-old animals.

On the other hand, our results show that insulin administration leads to a significant decrease in AMPK phosphorylation in all ages (Fig. 2F). In this case, there is a significant increase in the fold of response induced by insulin with age compared with ANOVA, being significantly higher in 12- than in 3-month-old mice.

As with insulin, to elucidate if leptin resistance is developed in the brainstem with age, we administered leptin via i.c.v. injection for 30 min in mice of 3-, 6-, and 12-month-old (Fig. 2A). In this case, we evaluated the phosphorylation of STAT3 and Akt as markers of leptin signaling. Leptin significantly increases STAT3 phosphorylation in 3- and 6-month-old animals compared with their respective saline group, but at 12 months of age, leptin fails to stimulate STAT3 phosphorylation (Fig. 2H). A significant effect of age was observed in the fold of response, with 6- and 12-month-old animals exhibiting significantly lower responses compared with 3-month-old animals. (Fig. 2I).

In the case of Akt, leptin significantly increases its phosphorylation at 3-month-old mice. No stimulation is observed in 6- and 12-month-old animals (Fig. 2J). Again, a significant decrease in response is observed with age (Fig. 2K).

Finally, a similar trend is observed for AMPK. Phosphorylation levels decrease at 3- and 6-month-old in response to leptin, but no effect is observed at 12-month-old animals (Fig. 2L), and as we can see in Fig. 2M, a significant age effect is observed when we analyze the fold of response.

### Insulin and Leptin Signaling Pathway in Brainstem with Age

**Fig. 3:**
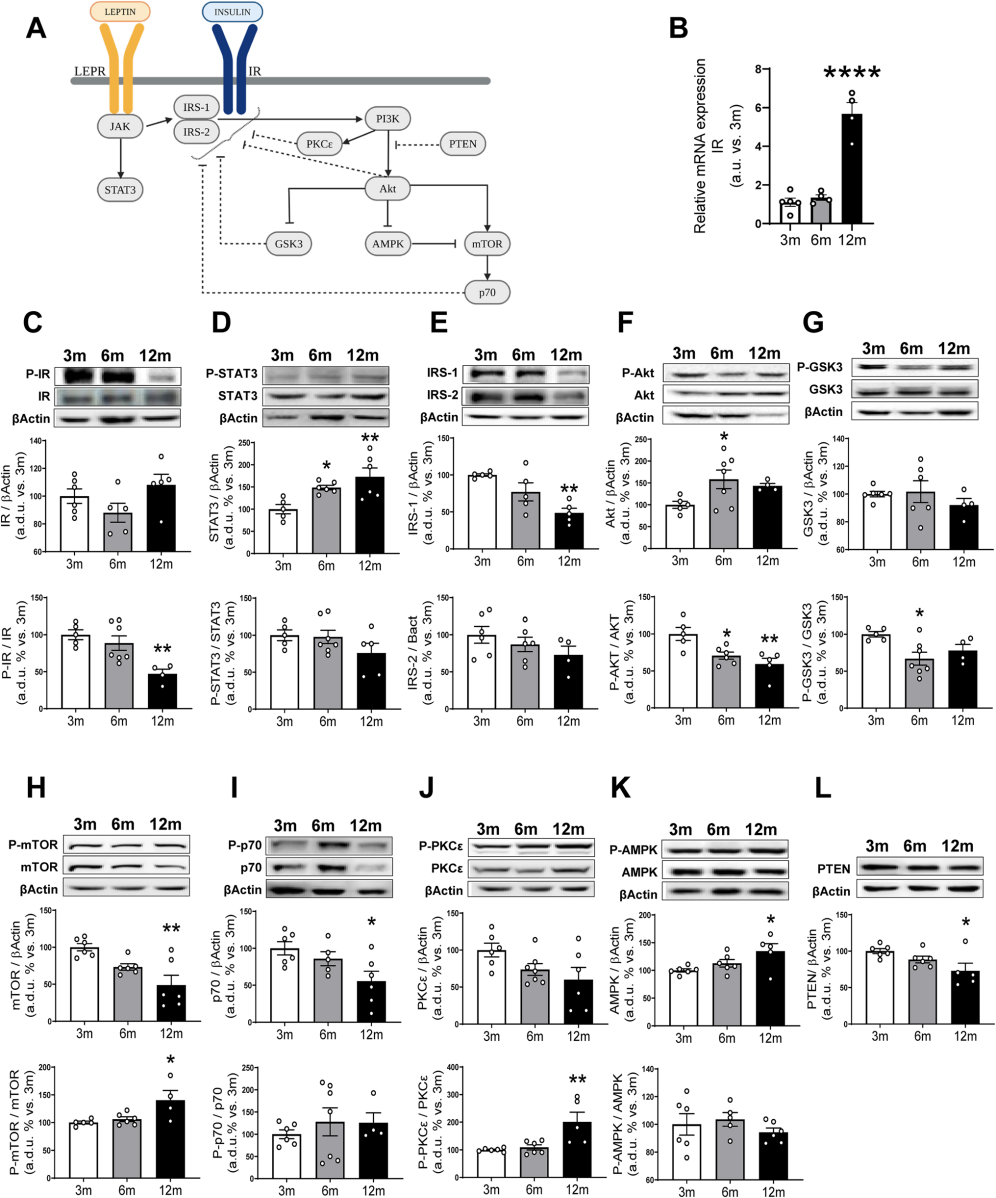
Metabolic sensors implicated in age-related changes in insulin and leptin signaling. **A** Schematic representation of the insulin and leptin signaling pathway. **B** Relative mRNA expression of IR in 3-, 6-, and 12-month-old mice from whole brainstem extracts. Densitometric evaluation was performed on several proteins from whole brainstem extract of 3-, 6-, and 12-month-old mice: **C** IR and its activation (P-IR/IR), **D** STAT3 and its activation (P-STAT3/STAT3), **E** IRS-1 and IRS-2, **F** AKT and its activation (P-AKT/AKT), **G** GSK3β and its activation (P-GSK3/GSK3), **H** mTOR and its activation (P-mTOR/mTOR), **I** p70 kinase and its activation (P-p70/p70), **J** PKCε and its activation (P-PKCε/PKCε), **K** AMPK and its activation (P-AMPK/AMPK), **L** PTEN. The upper panels display representative Western blots. Results are expressed as arbitrary units (a.u.) or arbitrary densitometric units (a.d.u.) relative to the 3-month-old group. Differences across ages were analyzed using one-way ANOVA followed by Dunnett’s post hoc test. *p < 0.05, **p < 0.01, ****p < 0.0001 compared to 3-month-old mice. Data are presented as means ± SEM of 4–6 animals per group

To understand the mechanism involved in the decreased insulin and leptin responsiveness, we studied possible alterations in several steps of their signaling pathway (Fig. 3A).

Downregulation and changes in basal phosphorylation of early components such as IR, STAT3, and IRSs have been related to their resistance at the central and peripheral levels. IR has been identified in the NTS, neighboring the DMV and in several other hindbrain regions [37]. Analysis of IR gene expression by RT-PCR shows a significant increase in 12-month-old animals (Fig. 3B). However, analysis of IR protein expression by Western blot shows no differences among the three groups of mice (Fig. 3C). Conversely, in 12-month-old animals, there is a significant decrease in their tyr-phosphorylation (Fig. 3C). In the case of STAT3, we observe a significant increase in protein levels with age, without changes in its basal phosphorylation (Fig. 3D). Finally, as shown in Fig. 3E, we can see a decrease in the levels of IRS-1 and IRS-2 protein expression with age, although only the decrease in IRS-1 reaches statistical differences.

Besides tyrosine phosphorylation, IR and IRSs can be negatively regulated by their phosphorylation in serine residues. Inhibitory sites are phosphorylated by several downstream serine kinases such as Akt, ERK1/2, GSK3, mTOR, and p70 and some members of the PKC family, as PKCε [48, 53].

In Fig. 3F–-J, protein expression and the phosphorylation levels of these different kinases related to the pathway can be seen. Regarding the Akt (Fig. 3F), although its protein expression increases with age, its phosphorylation decreases, suggesting reduced functionality despite the increased protein quantity. For the GSK3 (Fig. 3G), no variations in protein expression levels are observed between different ages, but there is a decrease in phosphorylation at 6- compared with 3-month-old animals. In the case of the mTOR (Fig. 3H), a decrease in protein expression with age and an increase in phosphorylation at 12- compared with 3-month-old animals is observed. The protein expression of p70 decreases with age while its phosphorylation does not change (Fig. 3I). Finally, the amount of PKCε tends to decrease with age, but its phosphorylation significantly increases in 12-month-old animals (Fig. 3J). No changes were detected in the protein expression or phosphorylation of the ERK1/2 protein (Supplementary Fig. 15).

Additionally, the AMPK shows an increase in protein expression at 12 compared with 3 months, although no differences were found in its phosphorylation (Fig. 3K). The analysis of the phosphatidylinositol 3,4,5-trisphosphate 3-phosphatase (PTEN), which counterbalances PI3K signaling by dephosphorylating specifically PIP3 (Fig. 3L), shows a decrease in protein expression levels at 12- compared with 3-month-old animals. Finally, no changes were observed in SOCS3 protein expression (Supplementary Fig. 16).

### Leptin Receptor in Brainstem with Age

**Fig. 4:**
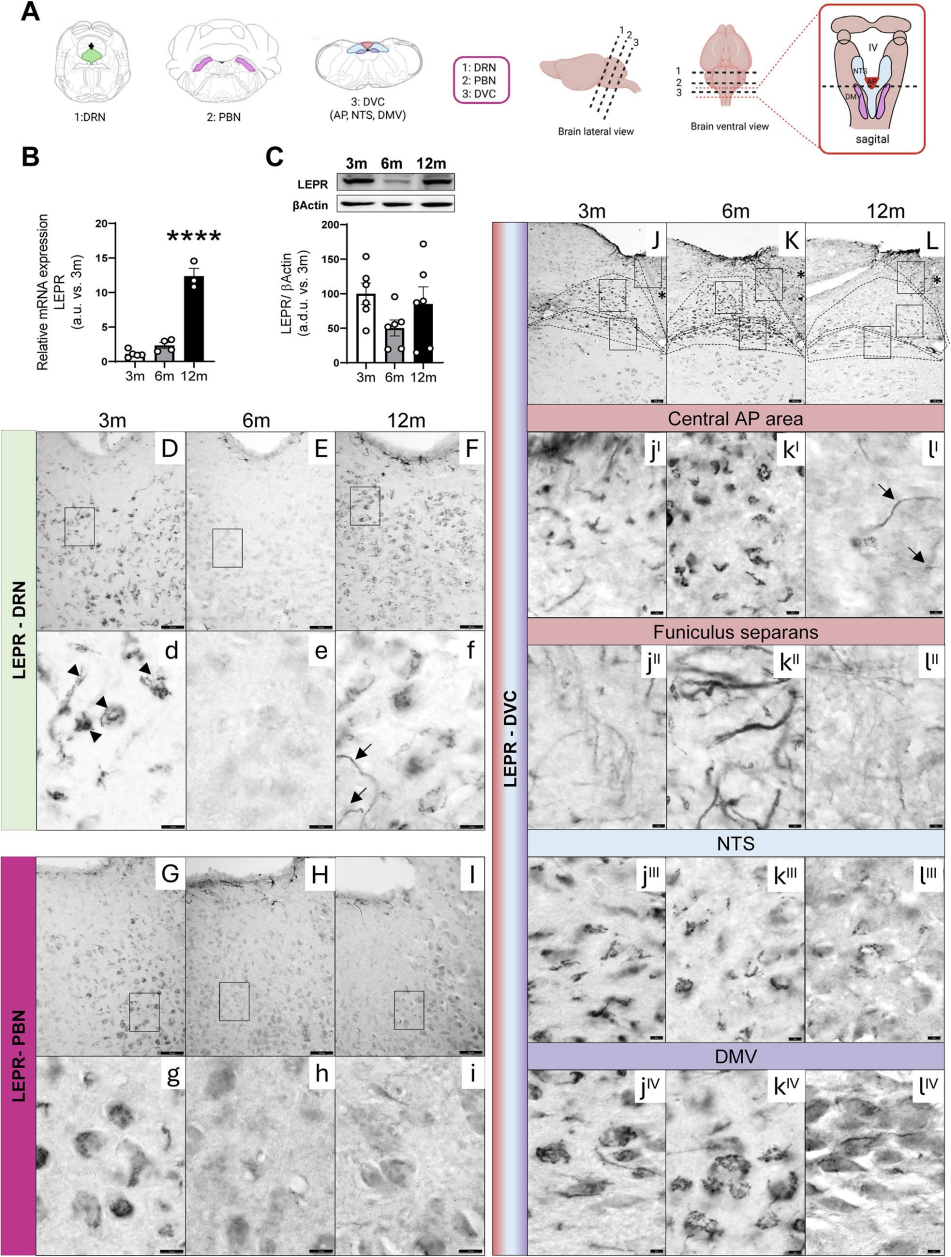
Age-dependent changes in LEPR expression. **A** Schematic drawings of mice brainstem nuclei showing the levels analyzed by immunohistochemistry: dorsal raphe nucleus (DRN), parabrachial nucleus (PBM) and dorsal vagal complex (DVC) comprising the area postrema (AP), nucleus of the solitary tract (NTS), and dorsal motor nucleus of the vagus (DMV). **B** Relative mRNA expression of LEPR in 3-, 6-, and 12-month-old mice from whole brainstem. **C** Densitometric evaluation of LEPR protein in whole brainstem extracts from 3-, 6-, and 12-month-old mice by Western blot. Results are expressed as arbitrary units (a.u.) or arbitrary densitometric units (a.d.u.) relative to 3-month-old mice. Differences across ages were analyzed using one-way ANOVA followed by Dunnett’s post hoc test. *p < 0.05, **p < 0.01, ***p < 0.001 compared with 3-month-old mice. Data are presented as means ± SEM of 4–6 animals per group. **D-l**^**IV**^ Immunohistochemical detection of LEPR in representative 25 µm coronal sections of DRN, PBN, and DVC from 3-, 6-, and 12-month-old mice. Low-magnification view of LEPR immunostaining distributed throughout DRN (**D–F**), PBN (**G–I**), and DVC (**J–L**) (scale bar equals 50 µm (**D–F, G–I**); scale bar equals 100 µm (**J–L**)). Black square indicates the ROIs shown at high magnification (scale bar equals 10 µm for **d–i** and 50 µm for **j**^**I**^**–l**^**IV**^) for comparison of immunolabeling between 3- (**d**), 6- (**e**), and 12-month **(f)**-old mice at the level of DRN, 3- (**g**), 6- (**h**), and 12-month **(i)**-old mice at the level of PBN and 3- (**j**^**I**^**–j**^**IV**^), 6- (**k**^**I**^**–k**^**IV**^), and 12-month (**l**^**I**^**–l**^**IV**^)-old mice in DVC. The immunoreaction was localized in the cytoplasm of the cells as a granular reaction resembling the Golgi apparatus **(**arrowheads in d**)**. Arrows point to glial processes. The central areas of AP **(**asterisks in J, K, and L**)** are shown at high magnification in **j**^**I**^**–l**^**I**^ where some processes are labeled (arrows). **J**^**II**^**–l**^**II**^ shows the glial process of the ventrolateral surface of the AP (the *funiculus separans*). **j**^**III**^**, k**^**III**^**, **and **l**^**III**^ represent NTS and (**j**^**IV**^, **k**^**IV**^), and(**l**^**IV**^) DMV. The figure shows a representative pattern reproduced in 2–3 animals per group

Functional leptin receptors have been described in several nuclei in the brainstem [66, 67]. Analysis of relative mRNA expression of LEPR in extracts of brainstem reveals higher levels in 12- compared with 3-month-old animals (Fig. 4B). When we analyzed LEPR protein expression by Western blot, there were no changes in 12- related to 3-month-old mice; however at 6 months of age, there is a tendency to decrease, although it does not reach statistical significance (Fig. 4C).

Immunohistochemical detection of LEPR in ROIs (Fig. 4A) using a polyclonal antiserum against a peptide mapping the carboxy terminus of LEPR is shown in Fig. 4D–L. Immunoreaction is localized in the cytoplasm of the cells as a granular reaction resembling the Golgi apparatus (arrowheads in Fig. 4d label some cells) as previously described [68, 69]. The immunoreaction is not uniform in all nuclei that we have explored. In DRN and PBN, immunostaining seems to decrease in 6-month-old animals (Fig. 4E and H and Supplementary Fig. 18 A), as well as the number of immunoreactive granules per cell as shown in higher magnification in Fig. 4e and h. In 12-month-old animals, the decrease in immunoreactivity in PBN (Fig. I and i) does not reach significance (Supplementary Fig. 18 A). In DRN (Fig. 4F and f), some processes are also seen (arrows in Fig. 4f).

Looking at DVC, at the level of AP, two different areas can be distinguished. On the one hand, it is the central area (asterisks in Fig. 4J-–L), where mainly cell bodies are labeled (Fig. 4j^I^–l^I^). In this central area, an apparent decrease is observed at 12 months of age (Fig. 4l^I^) although it does not reach significance (Supplementary Fig. 18 A) and only a few processes can be seen (arrows in Fig. 4l^I^). On the other hand, it is the interface between the AP and the NTS (*funiculus separans*) where the immunolabeling corresponds to glial processes which tend to increase in 6-month-old animals without reaching statistical significance (Fig. 4j^II^-l^II^). In the NTS, the immunolabeling tends to decrease with age (Fig. 4j^III^, k^III^, and l^III^ and Supplementary Fig. 18 A) However, in DMV, immunolabeled neurons are similar in 3-, 6-, and 12-month-old animals (Fig. 4 j^IV^, k^IV^, and l^IV^).

### Cytokines and Inflammatory Pathways in the Brainstem During with Age

Inflammation has been related to the development of insulin resistance. For this reason, we have analyzed some inflammation markers in the brainstem from mice of 3, 6, and 12 months of age.

**Fig. 5:**
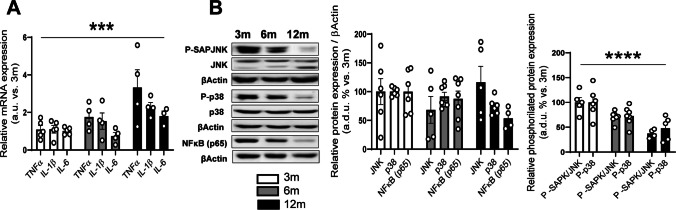
Signs of inflammation in the brainstem during aging. **A** Relative mRNA expression of the proinflammatory cytokines TNFα, IL-1β, IL-6 in 3-, 6-, and 12-month-old mice from whole brainstem extracts. **B** Densitometric evaluation of JNK and its activation (P-JNK/JNK), p38 and its activation (P-p38/p38), and NF-κBp65 protein in whole brainstem extracts from 3-, 6-, and 12-month-old mice. Results are expressed as arbitrary units (a.u.) or arbitrary densitometric units (a.d.u.) relative to the 3-month-old group. Differences in proinflammatory markers across ages were analyzed using two-way ANOVA followed by Dunnett’s post hoc test. *p < 0.05, **p < 0.01, ***p < 0.001 compared with 3-month-old mice. Data are presented as means ± SEM of 4–6 animals per group

As shown in Fig. 5A, relative mRNA expression of tumor necrosis factor alfa (TNFα) and interleukins (IL)−1β and IL-6 significantly increases with age. Additionally, we analyzed the protein expression and phosphorylation statuses of the serine kinases JNK, p38, and the protein expression of the transcription factor NFκB (p65). As we can see in Fig. 5B, there are no significant differences in the protein expression of the three proteins, although there is a tendency for p38 and NFκB (p65) to decrease in 12-month-old animals. Finally, the phosphorylation of JNK and p38 significantly decreases with age (Fig. 5B).

### Age-Dependent Expression of IBA 1 in the Brainstem

**Fig. 6:**
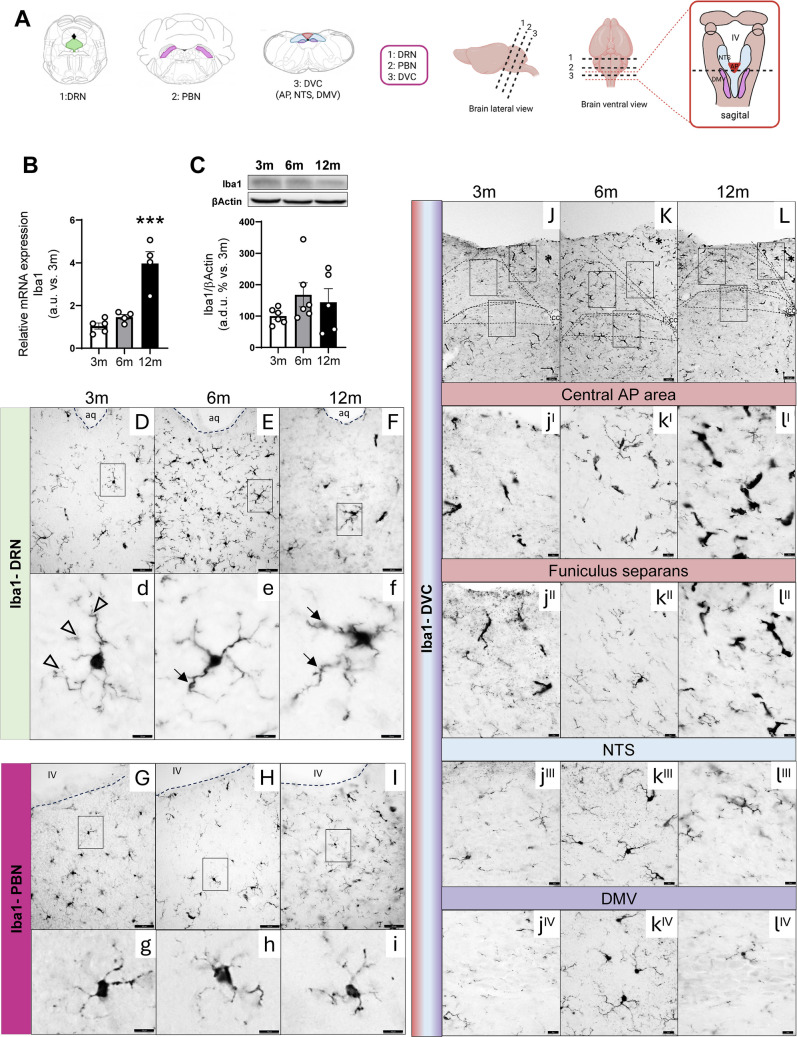
Age-dependent changes in Iba1 expression. **A** Schematic drawings of mouse brainstem nuclei showing the levels analyzed by immunohistochemistry: dorsal raphe nucleus (DRN), parabrachial nucleus (PBM), and dorsal vagal complex (DVC) comprising the area postrema (AP), nucleus of the solitary tract (NTS), and dorsal motor nucleus of the vagus (DMV). **B** Relative mRNA expression of the ionized calcium binding adaptor molecule 1 (Iba1), a constitutive identity marker of microglia, in 3-, 6-, and 12-month-old mice from whole brainstem. **C** Densitometric evaluation of Iba1 protein whole brainstem extracts from 3-, 6-, and 12-month-old mice by Western blot. Results are expressed as arbitrary units (a.u.) or arbitrary densitometric units (a.d.u.) relative to 3-month-old mice. Differences across ages were analyzed using one-way ANOVA followed by Dunnett’s post hoc test. *p < 0.05, **p < 0.01, ***p < 0.001 compared with 3-month-old mice. Data are presented as means ± SEM of 2 animals per group. **D-l**^**IV**^ Immunohistochemical detection of Iba1 in representative 25 µm coronal sections of DRN, PBN, and DVC from 3-, 6-, and 12-month-old mice. Low-magnification view of microglia distributed throughout DRN (**D–F**), PBN (**G–I**), and DVC (**J–L**) (scale bar equals 50 µm (**D–F, G–I**); scale bar equals 100 µm (**J–L**)). Black square indicates the ROIs shown at high magnification (scale bar equals 10 µm for **d–i** and 50 µm for **j**^**I**^**–l**^**IV**^) for comparison of microglial fine structure between 3- (**d**), 6- (**e**), and 12-month (**f**)-old mice at the level of DRN, 3- (**g**), 6- (**h**), and 12-month **(i**-old mice at the level of PBN and 3- (**j**^**I**^**–j**^**IV**^), 6- (**k**^**I**^**–k**^**IV**^), and 12-month (**l**^**I**^**–l**^**IV**^)-old mice in DVC**.** Small cell bodies and long slender ramifications **(**white arrowheads**)** in young (**d**) but progressively bigger cell bodies and short thick cell processes **(**black arrows**)** in aged animals (**e–f**). Fine immunolabeled structures in the neuropil between immunostained somas are observed frequently in young animals (arrows in d) in comparison to 12-month-old mice. In the PBN a slight increase in immunoreactivity is observed with aging (**I**) without notable morphological changes (**g–i**). In the DVC asterisks point to the AP in 3- (**J**), 6- (**K**), and 12-month-old animals (**L**). **j**^**I**^**–k**^**I**^ represent the central AP area and (**j**^**II**^**–k**^**II**^) the *funiculus separans*. NTS shown in (**j**^**III**^**–k**^**III**^) and DMV in (**j**^**IV**^**–k**^**IV**^). The figure shows a representative pattern reproduced in 2–3 animals per group

In order to explore microglia in the brainstem, we analyzed the expression and distribution by immunohistochemistry of Iba1 in ROIs (Fig. 6A) as a constitutive identity marker of microglia.

Relative mRNA expression of Iba1 increases significantly with age (Fig. 6B). Iba1 protein expression, analyzed by Western blot (Fig. 6C), increases in 6-month-old mice, although in this case it does not reach statistical significance.

At the level of DRN, immunostained microglia with Iba1 antibody seem to increase at 6 months of age (Fig. 6E) compared with 3- and 12-month-old animals (Fig. 6D, F and Supplementary Fig. 18B). Three-month-old mice have a smaller cell body and longer slender ramifications (Fig. 6d) than 6- and 12-month-old animals (Fig. 6e and f). Fine immunolabeled structures in the neuropil between immunostaining somas are more frequently observed in 3-month-old animals (arrows in Fig. 6d) compared with the 12-month-old animals (Fig. 6f), indicative of a distal arborization typical of “branched” or “resting” microglia (arrowhead). As mice age, microglia show progressively bigger cell bodies and shorter, thicker cell processes (arrowheads in Fig. 6e–f) characteristic of the transition from surveying microglia to a more reactive phenotype. At the level of PBN, decreased immunostaining is observed at 6 months (Fig. 6H) compared to 3- and 12-month-old animals (Fig. 6G, I and Supplementary Fig. 18B), with a cell morphology similar between the 3 ages (Fig. 6g-–i). However, at the level of DVC (Fig. 6J-–L), there is an increase with age (Supplementary Fig. 18B). In AP, immunostaining seems to increase, both in the central area (Fig. 6j^I^–l^I^, asterisks in Fig. 6J-–L) and in the *funiculus separans* (Fig. 6j^II^–l^II^ and Supplementary Fig. 18B) without reaching significance in *funiculus separans*. Finally, at the level of DMV (Fig. 6j^IV^–k^IV^), the immunolabeled cells seem to increase slightly in 6-month-old animals without changes at the level of NTS (Fig. 6j^III^–k^III^ and Supplementary Fig. 18B).

### GFAP Expression in the Brainstem with Age

GFAP is the main component of astrocyte intermediate filaments and has become a prototypical marker of reactive astrocytes [70].

**Fig. 7:**
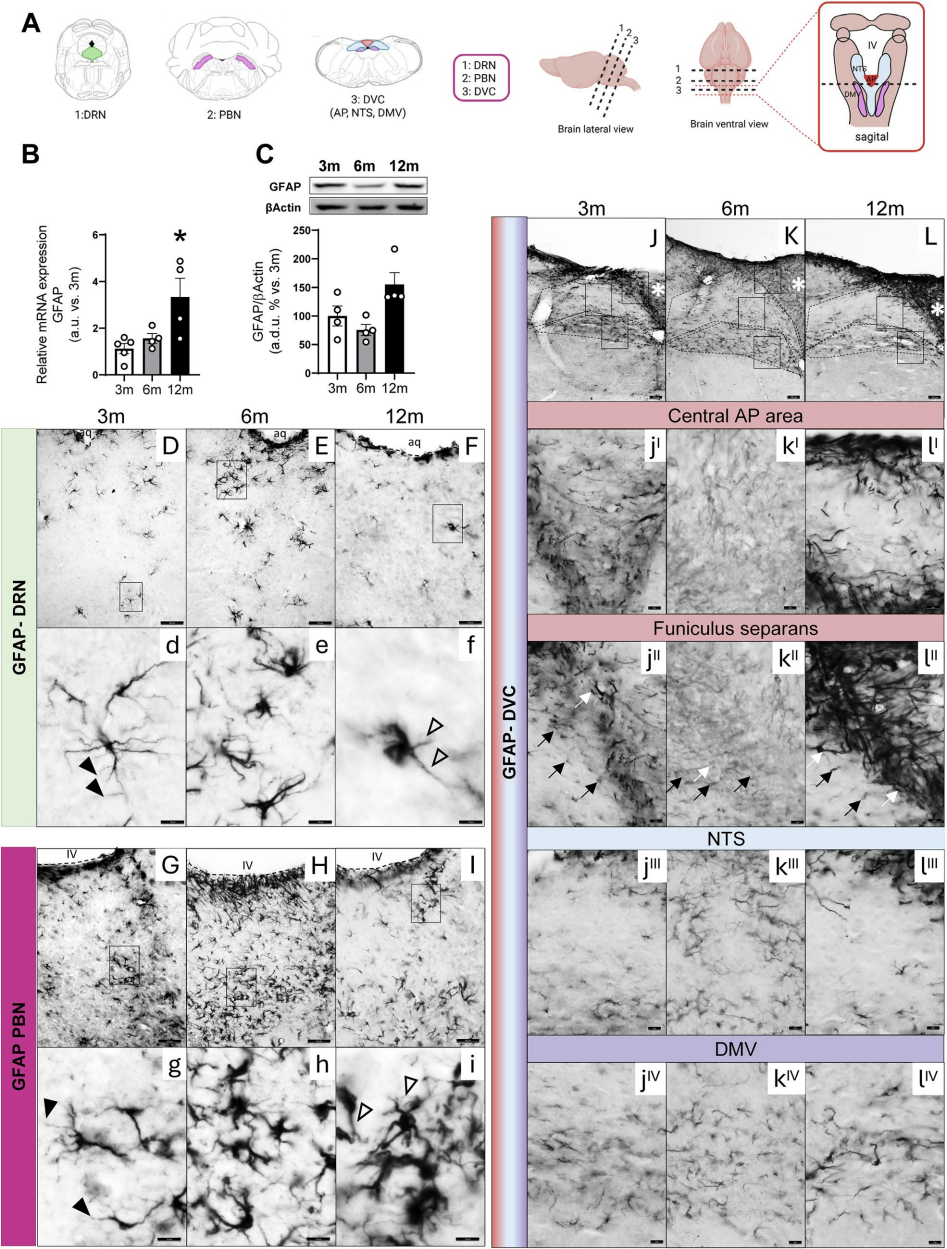
Age-dependent changes in GFAP expression. **A** Schematic drawings of mice brainstem nuclei showing the levels analyzed by immunohistochemistry: dorsal raphe nucleus (DRN), parabrachial nucleus (PBM) and dorsal vagal complex (DVC) comprising the area postrema (AP), nucleus of the solitary tract (NTS) and dorsal motor nucleus of the vagus (DMV). **B** Relative mRNA expression of GFAP in 3-, 6-, and 12-month-old mice from whole brainstem. **C** Densitometric evaluation of GFAP protein in whole brainstem extracts from 3-, 6-, and 12-month-old mice by Western blot. Results are expressed as arbitrary units (a.u.) or arbitrary densitometric units (a.d.u.) relative to 3-month-old mice. Differences across ages were analyzed using one-way ANOVA followed by Dunnett’s post hoc test. *p < 0.05, **p < 0.01, ***p < 0.001 compared with 3-month-old mice. Data are presented as means ± SEM of 4–6 animals per group. **D-l**^**IV**^ Immunohistochemical detection of GFAP in representative 25 µm coronal sections of DRN, PBN and DVC from 3-, 6-, and 12-month-old mice. Low-magnification view of GFAP immunostaining distributed throughout DRN (**D–F**), PBN (**G–I**), and DVC (**J–L**) (scale bar equals 50 µm (**D–F**,** G–I**); scale bar equals 100 µm (**J–L**)). Black square indicates the ROIs shown at high magnification (scale bar equals 10 µm for **d–i** and 50 µm for **j**^**I**^**–l**^**IV**^) for examination of astrocyte processes in 3- (**d**), 6- (**e**), and 12-month (**f**)-old mice at the level of DRN, 3- (**g**), 6- (**h**), and 12-month **(i)**-old mice at the level of PBN and 3- (**j**^**I**^**–j**^**IV**^), 6- (**k**^**I**^**–k**^**IV**^) and 12-month (**l**^**I**^**–l**^**IV**^)-old mice in DVC. GFAP-immunoreactive cells exhibit typical features of differentiated stellate astrocytes in DRN and PBN with apparently increased size and complexity in the transition from 3-month (**d **and **g**) to 12-month-old animals (**f **and **i**). Astrocyte processes in 3-month-old animals which remain separated into discrete areas have fine processes extending from the main cellular processes (black arrowheads in** d**) while being hypertrophic in 12-month-old animals (empty arrowheads in** f**). Few scattered overlapping processes and a dense fibrous network suggestive of the apparent formation of a syncytium of astrocytic processes develop from 6-month-old animals in PBN (**h–i**). GFAP immunolabeling in AP distinguishes two areas: a central area (white asterisks in **J–K**) and the *funiculus separans* the border zone between AP and NTS (**j**^**II**^**–l**^**II**^). Dense GFAP immunostaining in AP is confined mainly to the border but not within its central part. Long processes between AP and NTS (white arrows in **j**^**II**^**–l**^**II**^) and cross-sectioned processes (black arrows in** j**^**II**^**–l**^**II**^) seem to decrease in intensity at 6-month-old mice. In NTS a slight increase in immunoreactivity is observed in 6-month-old animals (**k**^**III**^and** k**^**IV**^). The figure shows a representative pattern reproduced in 2–3 animals per group

The analysis of GFAP is shown in Fig. 7 and Supplementary Fig. 18C. Relative mRNA expression of GFAP increases significantly in 12-month-old mice (Fig. 7B) which is followed by an increase in protein level, although in this case it does not reach statistical significance (Fig. 7C).

As early studies on astroglia noted both their morphological complexity and heterogeneity in different nuclei and conditions [71], we next analyzed the morphological pattern in DRN, PBN, and DVC nuclei (Fig. 7A) by GFAP immunohistochemistry on coronal sections of the brainstem (Fig. 7D-–L). In 3-month-old animals, immunostained astrocyte processes remain separated into discrete areas in the DRN (Fig. 7D) and PBN (Fig. 7G). The cellular body has fine processes extending from the main cellular processes (arrowheads in Fig. 7d and g) while being hypertrophic in 12-month-old animals (arrowheads in Fig. 7f and i). A few scattered overlapping processes and a dense fibrous network suggest the apparent formation of a syncytium of astrocytic processes that developed in 6- and 12-month-old mice (Fig. 7e, f in DRN and h, i in PBN).

GFAP immunoreactivity in the DVC revealed a striking pattern, which distinguished it from the surrounding brainstem nuclei. Within the DVC, a distinct subregional distribution of GFAP immunoreactivity was observed (Fig. 7J-–L). Weak GFAP immunostaining is observed in the central area of AP (asterisks in Fig. 7J-–L; 7j^I^–l^I^). A dot shape of GFAP immunoreactivity was selectively observed at the interface between the AP and NTS (Fig. 7j^II^–l^II^ blank arrows) which represents a cross-section of GFAP-positive processes. Also, some long processes between AP and NTS are observed (black arrows Fig. 7j^II^–l^II^). The immunostaining of the two kinds of processes seems to be more intense in 12- compared with 3-month-old animals (Fig. 7j^II^–l^II^ and Supplementary Fig. 18 C). NTS and the DMV appear more heavily labeled than AP. However, while in NTS the immunostaining decreases at 12 months (Fig. 7j^III^–l^III^ Supplementary Fig. 18 C), in DMV, it increases with age (Fig. 7j^IV^–l^IV^ Supplementary Fig. 18 C).

## Discussion

In this study, insulin or leptin was injected into the lateral cerebral ventricle of 3-, 6-, and 12-month-old male mice to determine whether age can influence their signaling pathways in the brainstem. As expected, an impaired response in both insulin and leptin appears with age, more specifically, in early aging. This insulin and leptin resistance in the brainstem with age is accompanied by the downregulation or changes in basal phosphorylation of early components of their signaling pathways such as IR, LEPR, and IRS-1. Moreover, the impairment of these hormones is associated with local signs of inflammation. The increase in proinflammatory cytokine expression, alterations in the expression of specific markers of microglia and astrocytes (Iba1 and GFAP, respectively), and changes in their morphology support a neuroinflammatory process in the brainstem, which could also mediate the age-associated insulin and leptin resistance in the brainstem.

This resistance could contribute to the progressive decline of homeostatic mechanisms observed during physiological aging and determine the development of obesity and impaired glucose homeostasis [72]. Consequently, it could play a role in the pathogenesis of age-related diseases and ultimately increase mortality and reduce lifespan [55].

It has been described that body weight increases gradually with aging, mostly due to an increase in adiposity [73]. In rodents, age is also a factor with substantial effect on metabolic parameters. Many studies have been carried out related to metabolic alterations along with aging in rodents with some discrepancies in the results. We used C57BL/6J mice, which have been particularly suitable for studying metabolic disease [74]. Our results, showing that the adiposity index increases paralleled the change in body weight and fat pads, are consistent with those previous reports, indicating that mice continued to gain fat up to 12 months of age (Table 4).

As previously described, these mice develop mild glucose intolerance associated with increased adiposity [75]. Our analysis reflects that aged mice have elevated plasma glucose and insulin levels. Hyperinsulinemia can be an effective compensatory mechanism that preserves insulin action in mild and moderate insulin resistance [76]. Also, 12-month-old mice present elevated HOMA-IR, pointing to a possible alteration in glucose homeostasis (Table 4).

A large variability in blood glucose and insulin responses during GTT has been previously reported in different strains of mice [71, 77–79]. Pioneer studies in mice have shown that aging promotes glucose intolerance, despite an increase in glucose-induced insulin secretion [80]. However other studies have shown insulin resistance in aged mice, although discrepant results have been reported for glucose tolerance [81] or insulin resistance in old mice [82]. The response to glucose or insulin administration could be affected by adiposity, as we observed in 12-month-old mice, and changes in body composition (Table 4). However, an optimal model devoid of changes in fat mass does not exist, [75]. Under these circumstances, an alteration in the MTTs (GTT, ITT, and PTT) was also expected. However, when we performed the tests (Fig. 1), the glycemic response tends to worsen with age, but we could not find significant differences between the ages analyzed. One possibility is that we have conducted the study up to 12 months, and in some studies, the alterations were observed from 16 months of age [83].

In agreement with previous observations [68], the increase in adiposity is associated with hyperleptinemia in aged animals (Table 4), which can be interpreted as a compensatory mechanism to overcome a state of impaired leptin action in the brain, in a similar way as hyperinsulinemia compensates for peripheral insulin resistance, as observed in aged animals. The fact that, in addition to elevated serum leptin levels, body weight and fat pad mass increase with age suggests the development of resistance to central leptin action in these animals at 12 months of age, as it has been widely described in humans and even in older animals [84, 85].

The data presented in this article demonstrates an impaired insulin and leptin transduction mechanism in the brainstem with age at the earliest steps of both signaling pathways (Fig. 2B and H). The decreased response to insulin, detected in terms of IR phosphorylation (Fig. 2C), and to leptin, as STAT3 phosphorylation (Fig. 2I), can be appreciated at 6 months and remains in 12-month-old mice.

From this point, insulin and leptin signaling cascades, instead of being completely independent, have an overlap of the signaling pathways at the level of PI3K [8], which has been mainly related to the insulin and leptin action on glucose metabolism in the CNS [45]. Our data support that downstream in the signaling pathway, there is an impairment of Akt phosphorylation in response to insulin and leptin from 6-month-old animals (Fig. 2E and K). The fact that ANOVA analysis does not show significance in the insulin response (Fig. 2E) could be explained by the great dispersion of data in 3-month-old animals, despite the fact that there is almost no response to insulin in 6- and 12-month-old animals, as occurs with leptin (Fig. 2K).

According to data in the literature, AMPK phosphorylation reflects its activation [86, 87]. In the hypothalamus, it is established that insulin and leptin inhibit AMPK, and this inhibition is essential for their anorexigenic and weight loss effects. Although there is no data in the literature that allows us to establish the role of AMPK in the brainstem, our findings show that both insulin and leptin decrease AMPK phosphorylation (Fig. 2F and L). While the magnitude of response to insulin progressively increases with age (Fig. 2G), in the case of leptin administration, 12-month-old animals do not respond (Fig. 2L). Basal AMPK protein expression increases in the brainstem with age, but its basal phosphorylation does not change (Fig. 3K). Taking together these data suggests a differential role of leptin and insulin in the regulation of AMPK in the brainstem. This different response with age is difficult to explain considering that the signaling of both hormones through the Akt pathway is reduced. However, it cannot be ruled out that these hormones may act on AMPK through additional and specific pathways.

Given that insulin and leptin signaling in the brainstem has been implicated in the regulation of both food intake and glucose homeostasis, it would be reasonable to expect that age-related changes in this signaling might affect both processes. However, our findings indicate that 12-month-old animals display hyperglycemia and increased body weight, despite no significant changes in total daily food intake (data not shown). Since the regulation of food intake is primarily mediated by these hormones acting in the hypothalamus, an attenuated brainstem response to insulin and leptin with age may impact glucose regulation and adiposity, with potential additional involvement of hypothalamic mechanisms.

Our data indicate that the insulin and leptin resistance in the brainstem could be explained, at least partially, by the impairment of their signal transduction pathway. The progressive decline of leptin-induced STAT3 could not be related to changes in its protein expression, which increase with age, or its basal phosphorylation, which remains unchanged (Fig. 4D), but it can be explained by the alterations observed in LEPR. In this regard, although the mRNA expression of LEPR significantly increases in 12-month-old animals (Fig. 4B), without any changes in protein expression, in 6-month-old mice, there is a tendency to decrease (Fig. 4C). By immunohistochemistry, although the intensity of the immunoreaction is not uniform in ROIs analyzed, it appears to decrease with age in most regions (Fig. 4 and Supplementary Fig. 18 A).

By the other side, the decrease in the insulin-induced IR phosphorylation is not correlated with its protein expression, which does not change with age studied by Western blot (Fig. 3C), even though IR mRNA expression is significantly upregulated in 12-month-old animals (Fig. 3B). The impaired IR response may be due to an imbalance between the basal IR protein expression, which does not change, and IR tyr-phosphorylation, which decreases with age (Fig. 3C).

These early alterations in insulin and leptin signaling may account for the reduced Akt activation in response to both hormones (Fig. 2E–K). However, the observed decrease in IRS-1 protein expression (Fig. 3E), along with the imbalance between phosphorylated and total Akt basal levels (Fig. 3F), indicated that additional mechanisms could also contribute. Specifically, reduced Akt phosphorylation despite increased total Akt protein suggests impaired signaling functionality.

Inhibitory IR and IRS serine phosphorylation has been shown to be mediated by different serine/threonine kinases. As basal protein expression or phosphorylation of GSK3, p70 (Fig. 3G and I), and ERK1/2 (Supplementary Fig. 15) was unchanged or decreased, our results rule out the involvement of this protein as a mechanism involved in brainstem insulin and leptin resistance. The mTOR pathway is one of the branches that has been related to the development of insulin resistance [88]. The reduction in basal expression and the increase in its phosphorylation do not allow us to clarify possible changes in its activity with age. A similar pattern is observed in PKCε (Fig. 3J). Finally, basal PTEN protein expression decreases in 12-month-old animals (Fig. 3L), while SOCS3 levels remain unchanged (Supplementary Fig. 16), ruling them out as a potential mechanism underlying the age-related decrease in insulin or leptin response in the brainstem.

The higher expression of proinflammatory cytokines that we observe may support the idea that inflammation contributes to the decreased insulin and leptin response (Fig. 5A). However, our results do not show enhanced activation of the inflammatory pathway, as indicated by the fact that the basal protein expression of JNK, p38, and NFκB does not change with age, while the phosphorylation of JNK and p38 significantly decreases (Fig. 5B). Other models of central insulin resistance, such as mice under a high-fat diet (HFD) [89, 90] and aged Wistar rats [91], have shown signs of hypothalamic inflammation. More recently, it has been described that HFD increases the expression of proinflammatory cytokines in the brainstem of rats [92] and causes inflammation and insulin resistance in the brainstem of mice [64]. These data suggest that the mechanisms underlying central resistance to leptin and/or insulin could differ between HFD and aging. In addition, age-related alterations in insulin and leptin signaling may influence the interpretation of findings from HFD models, depending on the age of the animals used. For that reason, when conducting studies involving HFD in animal models, it is therefore essential to take into account the age of the animals as it can significantly affect metabolic responses, susceptibility to diet-induced changes, and overall physiological outcomes.

Nevertheless, subtle changes in cytokine expression and in glial cell markers and morphology point toward a progressive, low-grade inflammatory state with the development of microgliosis and astrogliosis associated with age. (Fig. 6, 7, and Supplementary Fig. 18B y 18 C). This neuroinflammatory milieu, while not overtly driven by classical inflammatory cascades, may contribute to age-related functional decline in brainstem-regulated processes. Our results agree with previous research that demonstrated that aging is associated with a chronic inflammatory state both in the periphery and in the CNS [93], accompanied by an impairment of the function of the immune system affecting both the innate and adaptive responses [94]. In the aging brain, microglia as well as other glial cells have a series of functional impairments that contribute to sustained activation, maintaining the chronic neuroinflammatory state [95]. These findings underscore the complexity of neuroinflammation in aging and highlight the need for further investigation into inflammatory mechanisms that may be at play.

Although the main effects of insulin and leptin in regulating energy balance and metabolism in the brainstem have been associated with neurons, we cannot rule out the involvement of glial cells. As our signaling data are from whole brainstem samples, at least in part, the effect may be due to glial cells. These interpretations, however, require further functional validation to confirm the brainstem cellular subtype and nuclei implicated.

The process of microglial transformation from resting to activated states is accompanied by marked morphological changes with several intermediate stages [96–98] which also become evident during aging and HFD [98–101]. In the hippocampus of aged mice, the significant differences in cell morphology are not followed by Iba1 mRNA level changes [102]. Although our immunohistochemical data reveal only modest morphological changes in Iba1-positive microglia (Fig. 6), such alterations are not definitive indicators of functional status. However, the observed presence of these cells, combined with a general trend toward increased Iba1 protein levels and a significant upregulation of its mRNA expression with age, strongly suggests a disruption in microglia-mediated maintenance of brain homeostasis. These findings point to a potential decline in the neuroprotective functions of microglia, which may contribute to the broader neuroinflammatory landscape in the brainstem associated with aging [101].

Previous studies have reported increased microglial activation in the NTS [103] and DMV supporting the involvement of the DVC in both normal and pathological regulation of energy balance [104]. Our findings further reinforce this role, particularly in the context of aging, by demonstrating age-associated changes in glial markers within the brainstem. As illustrated in Fig. 6k^III−IV^–l^III−IV^, these alterations suggest that age may compromise its homeostatic functions through glial-mediated mechanisms.

On the other side, it has been described that in healthy aging, the number of astrocytes remains unchanged, while the level of GFAP mRNA and protein increases in several brain regions in rodents and humans [105]. On the other hand, increasing evidence highlights the heterogeneity of astrocytes during aging, as well as across different brain regions. This variability has been particularly well characterized in the cortex and hippocampus, where distinct astrocyte subpopulations exhibit region-specific transcriptional profiles, morphologies, and functional responses [106]. Our results indicate that GFAP immunostaining distribution within the DVC revealed strong heterogeneity. Previous studies have demonstrated robust GFAP immunoreactivity in the NTS and DMN, whereas the AP exhibits comparatively weak labeling [107]. This regional pattern of GFAP expression is preserved throughout age, and most astrocytes in young animals display a differentiated stellate morphology, while in aged animals, these cells exhibit hypertrophic features consistent with a reactive or activated astrocytic phenotype.

Astrocytes in the NTS have been identified as primary glucosensors, playing a crucial role in detecting hypoglycemia and activating hindbrain neurons to initiate counter-regulatory responses [108, 109]. In our study, signaling data were derived from whole brainstem samples, which limits our ability to precisely attribute the observed changes to specific cell types or nuclei. Nonetheless, our results indicate that key signaling pathways are altered in the aged brainstem, suggesting a broader dysregulation of metabolic sensing and neuroglial communication. Future studies employing cell type-specific and regionally targeted approaches will be necessary to dissect the contributions of distinct brainstem populations, particularly astrocytes in the NTS, to age-related impairments in glucose homeostasis.

The present study, however, has a few limitations. Our interest is to find first early alterations for a middle-age range in healthy 12-month-old mice that may be susceptible to benefit from some pharmacologic or dietary treatment. Considering that animals in the final stage (from 24 months onward) may present age-related pathologies and have a reduced life expectancy, the feasibility of any potential intervention is limited. Nevertheless, gaining deeper knowledge about the oldest animals remains essential to confirm the progression and impact of these findings. Our work demonstrates insulin and leptin resistance with age in male mice. This represents a bias in our research, as it cannot be generalized to female mice, which also represents an area for future research. Finally, we have used whole extracts for the analysis of mRNA and protein. These interpretations, therefore, require further functional validation to confirm the brainstem cellular subtype and nuclei implicated. The complexity of neuroinflammation in aging highlights the need for further research into the inflammatory mechanisms that may be at play.

## Conclusion

Considering the findings of this study, we report for the first time the development of insulin and leptin resistance in the mouse brainstem until 12-month-old animals. These resistances are accompanied by alterations of early components of their signaling pathways. Moreover, the increase in proinflammatory cytokine expression, alterations in the expression of specific markers of microglia and astrocytes, and changes in their morphology support neuroinflammatory progress in the brainstem with age. Given that insulin and leptin signals from peripheral organs modulate glucose and metabolic homeostasis within the brainstem, the resistance of these hormones may contribute to the progressive decline of homeostatic mechanisms observed during physiological aging. This dysfunction could underlie the development of obesity and impaired glucose homeostasis and, consequently, may play a role in the pathogenesis of age-associated diseases. As the global population continues to age, understanding the molecular and cellular mechanisms underlying brainstem aging becomes increasingly important, due to the essential functions this region performs. This knowledge could pave the way for developing targeted interventions to maintain brainstem function and prevent age-related disorders.

## Supplementary Information

Below is the link to the electronic supplementary material.

ESM 1 Supplementary Material 1 (DOCX 4.66 MB)

## Data Availability

No datasets were generated or analysed during the current study.

## Abbreviations

AP: Area postrema
Akt: Protein kinase B
AMPK: AMP-activated protein kinase
BAT: Brown adipose tissue
CNS: Central nervous system
DMV: Dorsal motor nucleus of the vagus
DRN: Dorsal raphe nucleus
DVC: Dorsal vagal complex
ERK1/2: Extracellular signal-regulated protein kinase ½
eWAT: Epididymal white adipose tissue
GFAP: Glial fibrillar acid protein
GTT: Glucose tolerance test
GSK3: Glycogen-synthase-kinase-3
HFD: High-fat diet
HOMA-IR: Homeostasis model assessment of insulin resistance index
Iba1: Ionized calcium binding adaptor molecule 1
i.c.v.: Intracerebroventricular
IL-1β: Interleukin-1β
IL-6: Interleukin-6
INS: Insulin
i.p.: Intraperitoneal
IR: Insulin receptor
IRS-1: Insulin receptor substrates-1
IRS-2: Insulin receptor substrates-2
ITT: Insulin tolerance test
JAK: Janus kinase 2/signal transducer
JNK: C-Janus N-terminal kinase
LEPR: Leptin receptor
MAPK: Mitogen-activated protein kinase
mTOR: Mammalian targets of rapamycin
MTTs: Metabolic tolerance test
NF-κB: Nuclear factor-κB
NTS: Nucleus of the solitary tract
PBN: Parabrachial nucleus
PI3K: Phosphatidylinositol 3-kinase
PKCε: Protein kinase C-ε
PTT: Pyruvate tolerance test
pWAT: Perirenal white adipose tissue
p38: P38 mitogen-activated protein kinase
p70: Protein kinase p70S6
ROIs: Regions of interest
SOCS3: Suppressor of cytokine signaling 3
STAT3: Activator of transcription 3
sWAT: Inguinal subcutaneous white adipose tissue
TNFα: Tumor necrosis factor alpha
WATs: White adipose tissues
