# Supplementary material for: Development of Insulin and Leptin Resistance in the Mouse Brainstem with Age
Source: Mol Neurobiol. 2026 Jan 16;63(1):367. doi: 10.1007/s12035-025-05392-5 (PMC12811305; doi:10.1007/s12035-025-05392-5)
Supplement: Supplementary file 1 — Supplementary Material 1 (DOCX 4.66 MB) [file 12035_2025_5392_MOESM1_ESM.docx]

**Supplementary material for**

Development of insulin and leptin resistance in the mouse brainstem with age

**Author information**

Elvira De Frutos González^1^, Nuria Lauzurica^1^, Jose Joaquín Ochoa Navarro^1^, Miriam García San Frutos^1^, Fernando Aguado^2,3^, Teresa Fernández-Agulló^1^

1 Area of Physiology, Faculty Health Sciences, King Juan Carlos University, Alcorcón, Madrid, Spain.

2 Department of Cell Biology, Physiology and Immunology, Faculty of Biology, University of Barcelona, Barcelona, Spain.

3 Institute of Neurosciences, University of Barcelona, Barcelona, Spain.

**Corresponding author**

Miriam García San Frutos: miriam.garcia@urjc.es

Nuria Lauzurica: [nuria.lauzurica@urjc.es](mailto:nuria.lauzurica@urjc.es)

**Contents**

**Supplementary Fig. 1 Schematic representation of the procedure with**

**sectioned membranes, exemplified by i.c.v. experiments…………………………….2**

**Supplementary Fig. 2 Full uncropped blots image of figure 2B.…………….………2**

**Supplementary Fig. 3 Full uncropped blots image of figure 2D.……………………..3**

**Supplementary Fig. 4 Full uncropped blots image of figure 2F.……………………..4**

**Supplementary Fig. 5 Full uncropped blots image of figure 2H.……………………..5**

**Supplementary Fig. 6 Full uncropped blots image of figure 2J.……………………..6**

**Supplementary Fig. 7 Full uncropped blots image of figure 2L.……………………..7**

**Supplementary Fig. 8 Full uncropped blots image of figure 3C-G…………………...8**

**Supplementary Fig. 9 Full uncropped blots image of figure 3H-J………….………..9**

**Supplementary Fig. 10 Full uncropped blots image of figure 3K-L………….……..10**

**Supplementary Fig. 11 Full uncropped blots image of figure 4C…………….…..…10**

**Supplementary Fig. 12 Full uncropped blots image of figure 5B…………………..11**

**Supplementary Fig. 13 Full uncropped blots image of figure 6C…………………...12**

**Supplementary Fig. 14 Full uncropped blots image of figure 7C…………………...12**

**Supplementary Fig. 15 ERK1/2 and its activation (P-ERK1/2 / ERK1/2)**

**protein expression……………………………………………………….…...………..13**

**Supplementary Fig. 16 SOCS3 protein expression……………….…………………14**

**Supplementary Fig. 17 βActin protein expression…………………………………..15**

**Supplementary Fig. 18 Semiquantitative analysis of LEPR, Iba1 and GFAP……...16**

**Supplementary Table 1. F-statistics and p-values used in one-way ANOVA test analyses corresponding to Table 1………………………………………………….…17**

**Supplementary Table 2. Statistical values obtained from the results of each figure………………………………………………………………………………..18-19**

**Supplementary Table 3. Statistical values obtained by one-way ANOVA test analyses from the results of each IHC figure………………………………………....20**


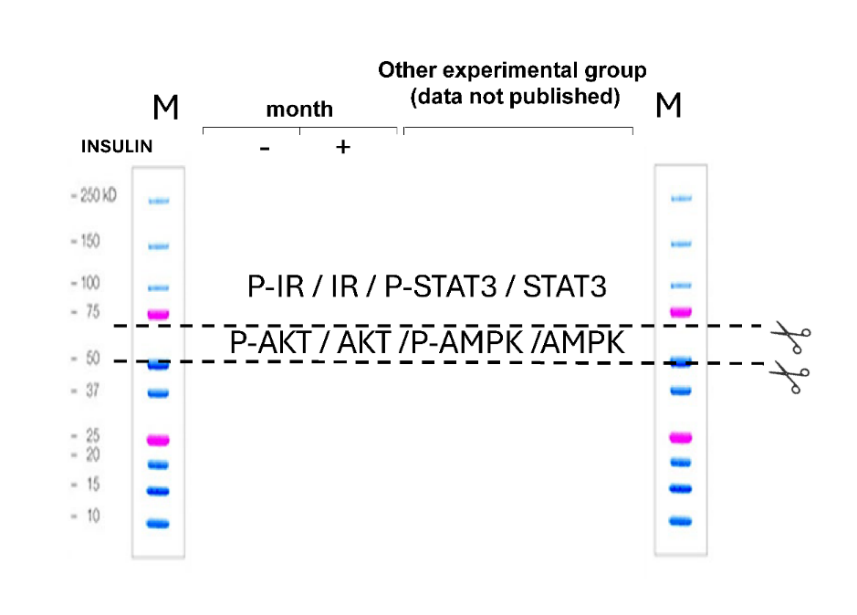


**Supplementary Fig. 1 Schematic representation of the procedure with sectioned membranes, exemplified by i.c.v. experiments**. To reduce animal usage and reagent consumption, membranes were sectioned according to the molecular weight of the proteins of interest. Membrane fragments from different gels were simultaneously incubated using the same primary and secondary antibody. M, molecular weight marker.


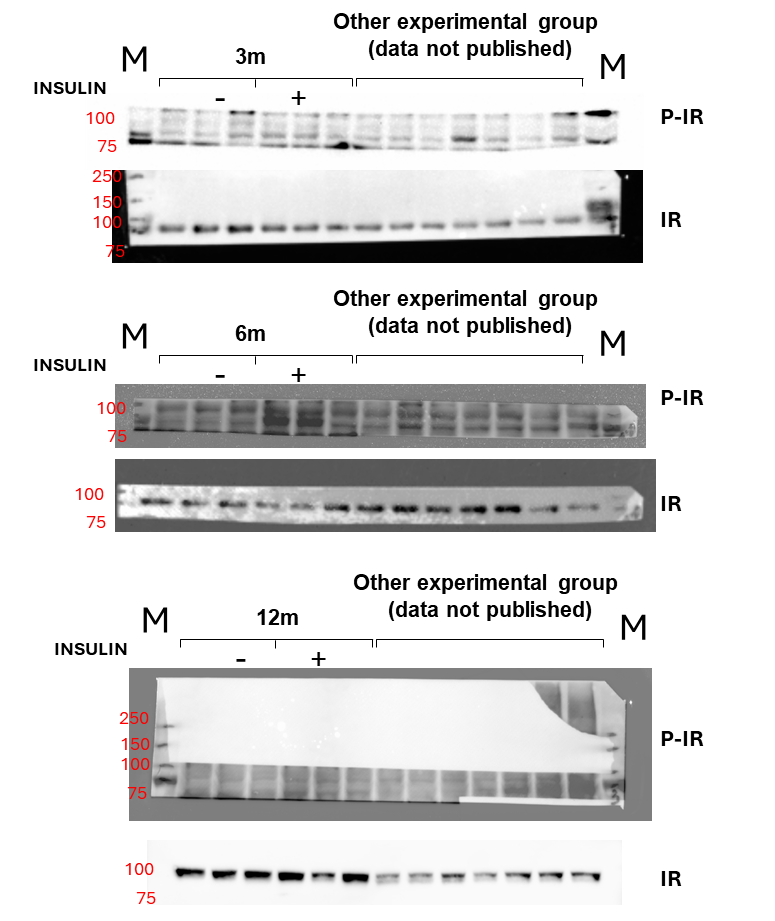


**Supplementary Fig. 2 Full uncropped blots image of figure 2B.** The membranes were sectioned according to the molecular weight of the proteins of interest. Membrane fragments from different gels were simultaneously incubated using the same primary and secondary antibody. During the P-IR immunodetection, membranes were covered with aluminum foil to prevent high-intensity nonspecific bands from interfering with the detection of the target band. M, molecular weight marker.


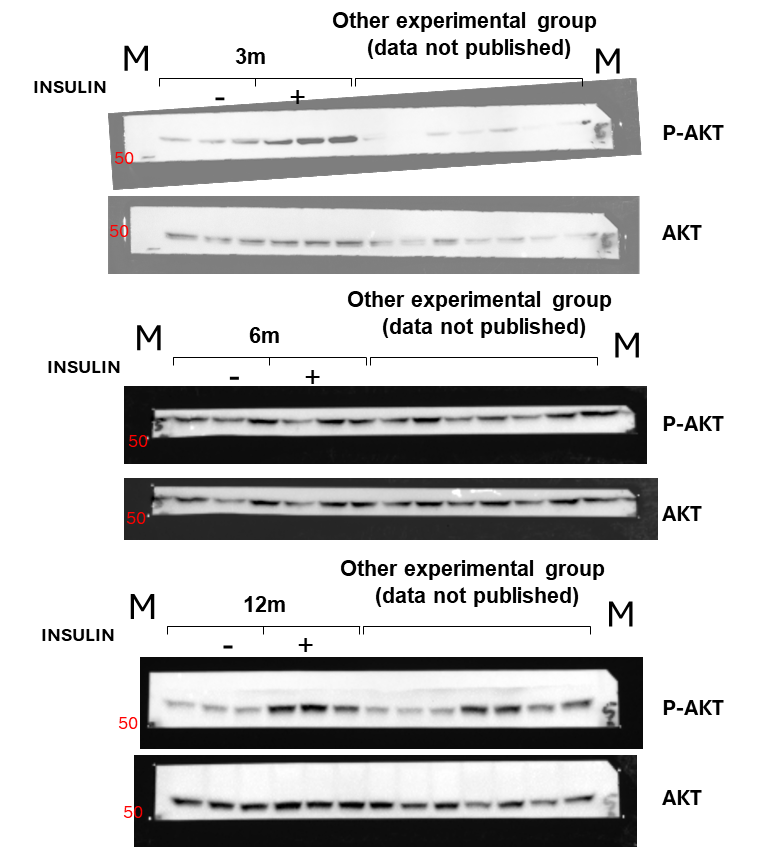


**Supplementary Fig. 3 Full uncropped blots image of figure 2D.** The membranes were sectioned according to the molecular weight of the proteins of interest. Membrane fragments from different gels were simultaneously incubated using the same primary and secondary antibody. M, molecular weight marker.


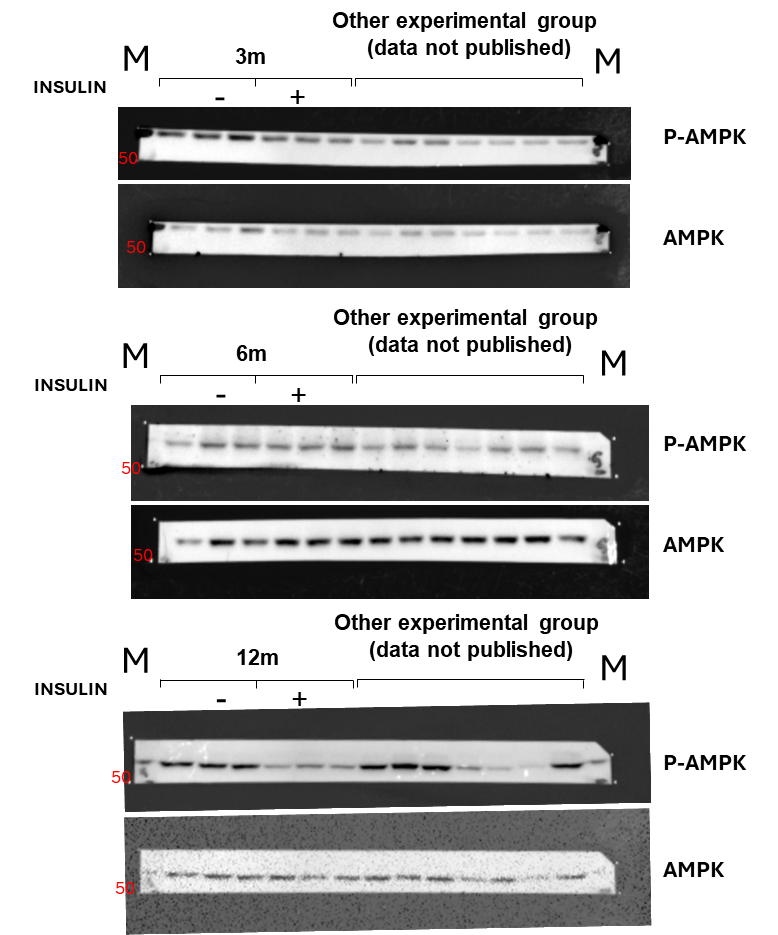


**Supplementary Fig. 4 Full uncropped blots image of figure 2F**. The membranes were sectioned according to the molecular weight of the proteins of interest. Membrane fragments from different gels were simultaneously incubated using the same primary and secondary antibody. M, molecular weight marker.


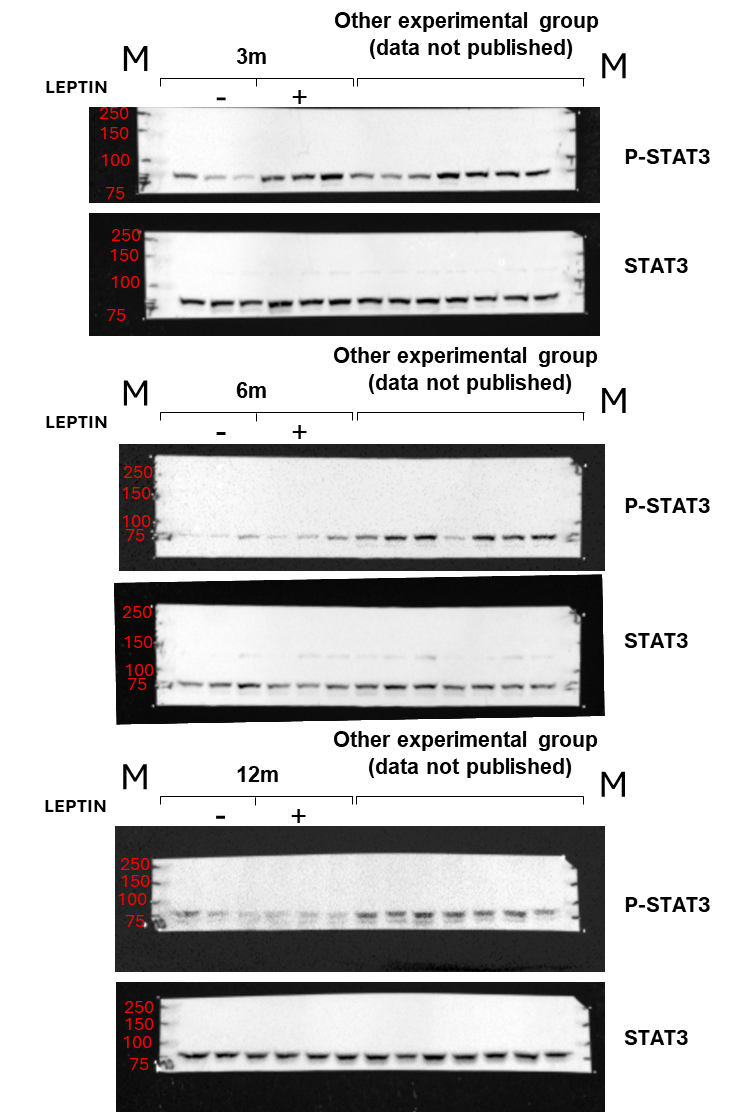


**Supplementary Fig. 5 Full uncropped blots image of figure 2H**. The membranes were sectioned according to the molecular weight of the proteins of interest. Membrane fragments from different gels were simultaneously incubated using the same primary and secondary antibody. M, molecular weight marker.


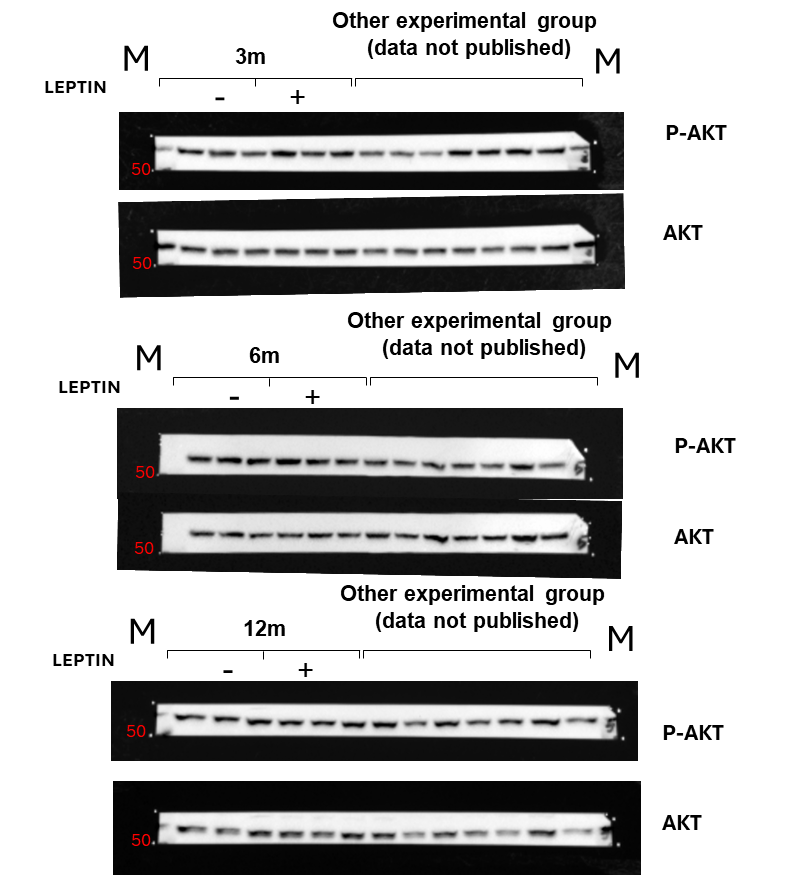


**Supplementary Fig. 6 Full uncropped blots image of figure 2J**. The membranes were sectioned according to the molecular weight of the proteins of interest. Membrane fragments from different gels were simultaneously incubated using the same primary and secondary antibody. M, molecular weight marker.


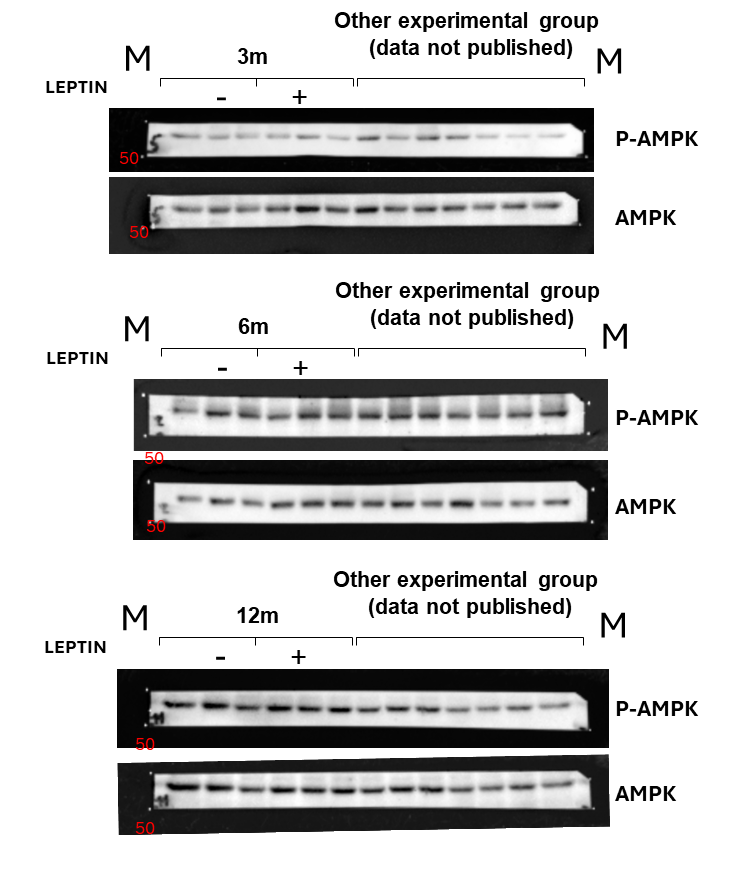


**Supplementary Fig. 7 Full uncropped blots image of figure 2L**. The membranes were sectioned according to the molecular weight of the proteins of interest. Membrane fragments from different gels were simultaneously incubated using the same primary and secondary antibody. M, molecular weight marker.


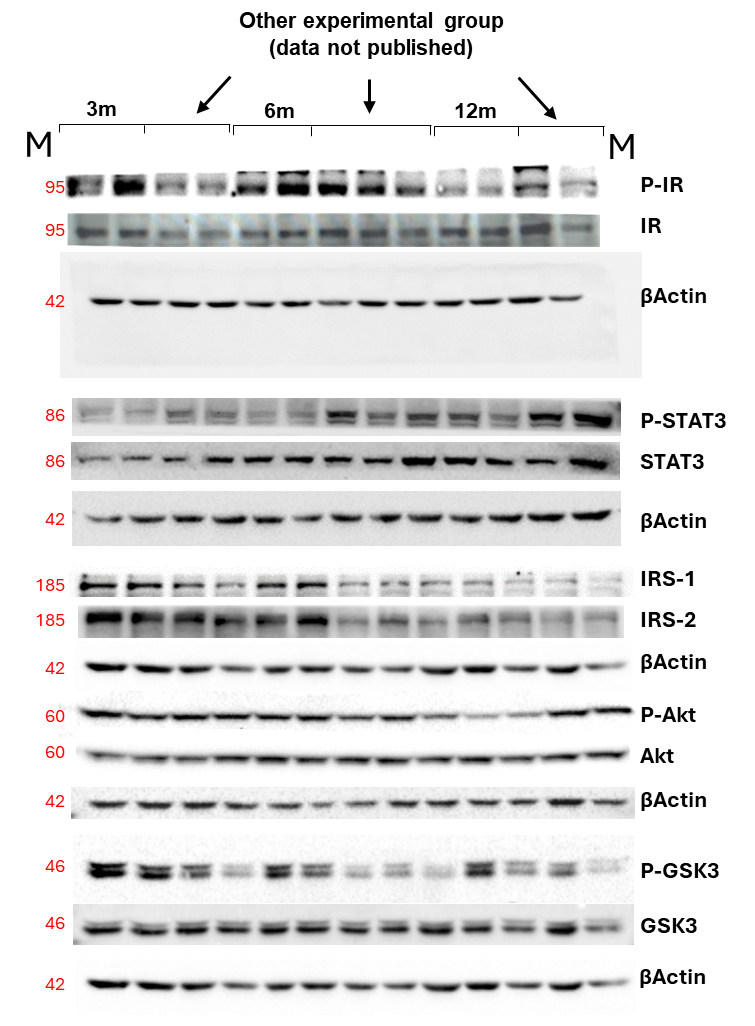


**Supplementary Fig. 8 Full uncropped blots image of figure 3C-G**. The membranes were sectioned according to the molecular weight of the proteins of interest. Membrane fragments from different gels were simultaneously incubated using the same primary and secondary antibody. M, molecular weight marker.


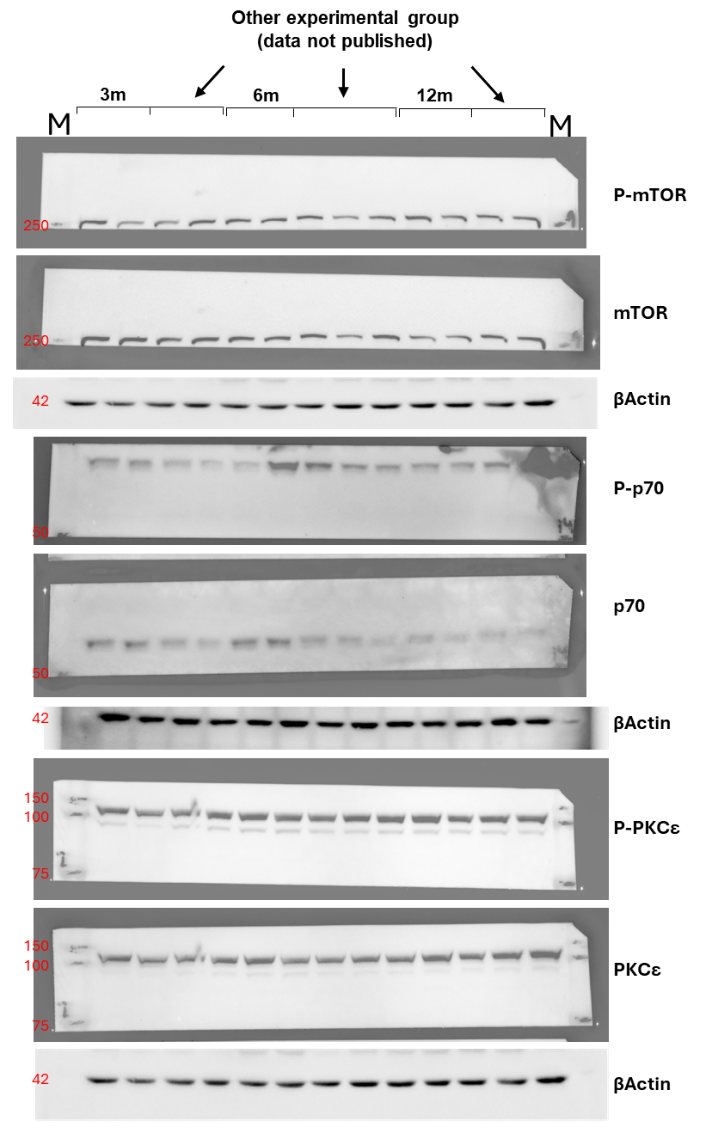


**Supplementary Fig. 9 Full uncropped blots image of figure 3H-J**. The membranes were sectioned according to the molecular weight of the proteins of interest. Membrane fragments from different gels were simultaneously incubated using the same primary and secondary antibody. M, molecular weight marker.


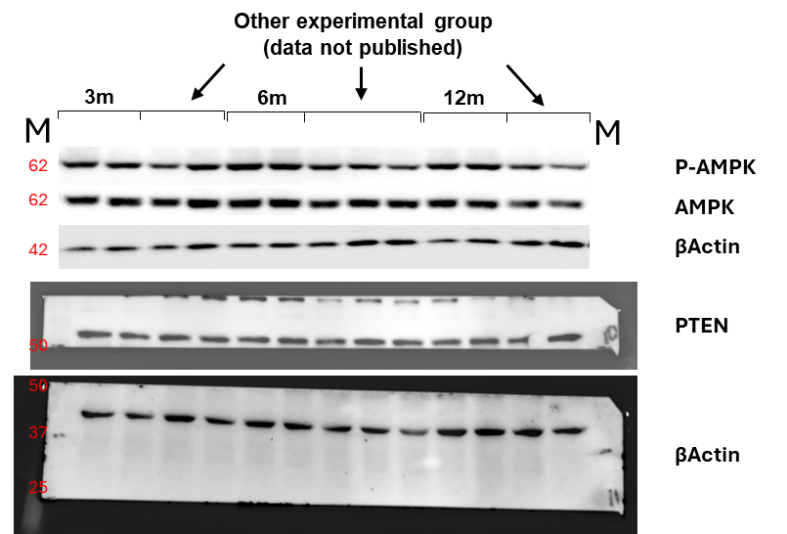


**Supplementary Fig. 10 Full uncropped blots image of figure 3K-L**. The membranes were sectioned according to the molecular weight of the proteins of interest. Membrane fragments from different gels were simultaneously incubated using the same primary and secondary antibody. M, molecular weight marker.


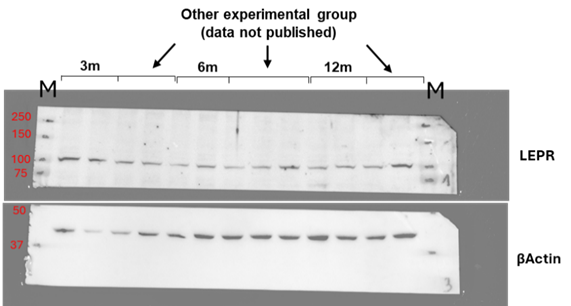


**Supplementary Fig. 11 Full uncropped blots image of figure 4C**. The membranes were sectioned according to the molecular weight of the proteins of interest. Membrane fragments from different gels were simultaneously incubated using the same primary and secondary antibody. M, molecular weight marker.


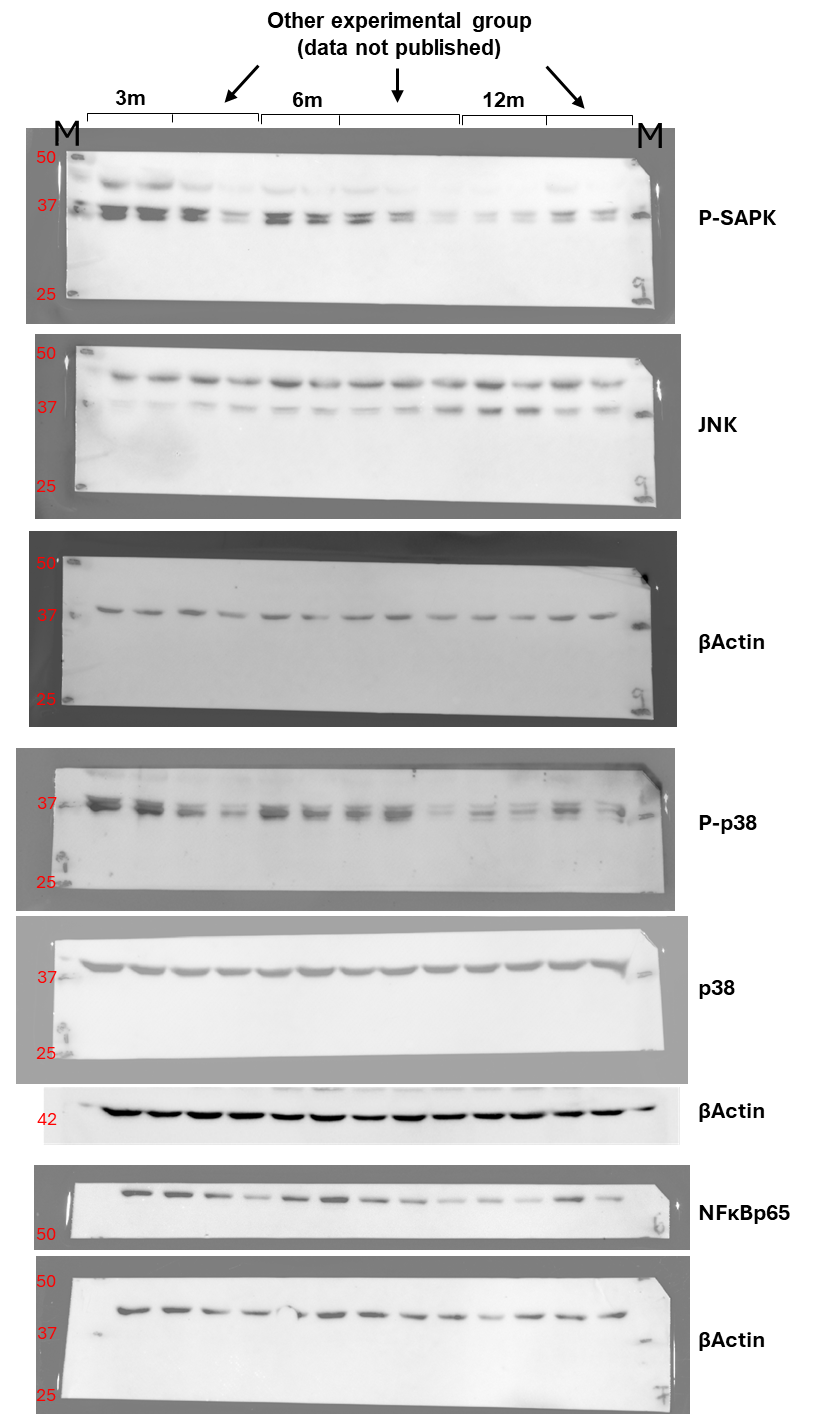


**Supplementary Fig. 12 Full uncropped blots image of figure 5B**. The membranes were sectioned according to the molecular weight of the proteins of interest. Membrane fragments from different gels were simultaneously incubated using the same primary and secondary antibody. M, molecular weight marker.


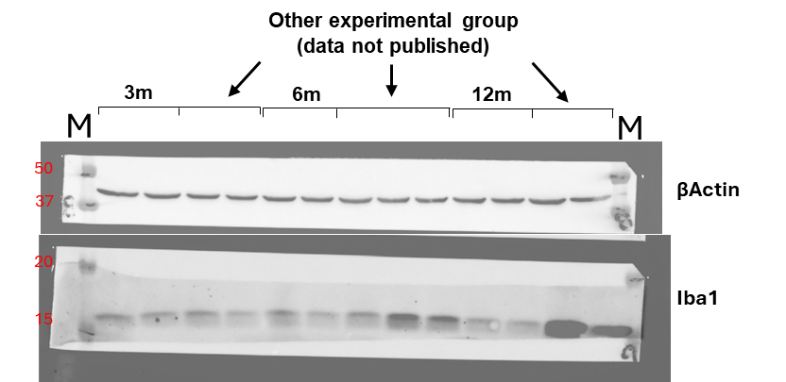


**Supplementary Fig. 13 Full uncropped blots image of figure 6C**. The membranes were sectioned according to the molecular weight of the proteins of interest. Membrane fragments from different gels were simultaneously incubated using the same primary and secondary antibody. M, molecular weight marker.


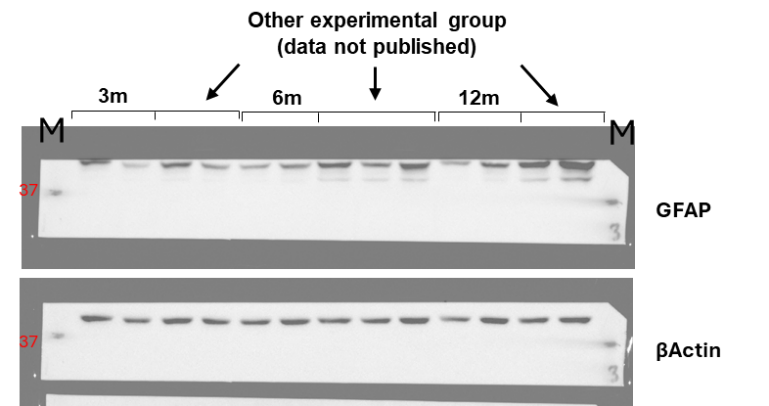


**Supplementary Fig. 14** **Full uncropped blots image of figure 7C**. The membranes were sectioned according to the molecular weight of the proteins of interest. Membrane fragments from different gels were simultaneously incubated using the same primary and secondary antibody. M, molecular weight marker.


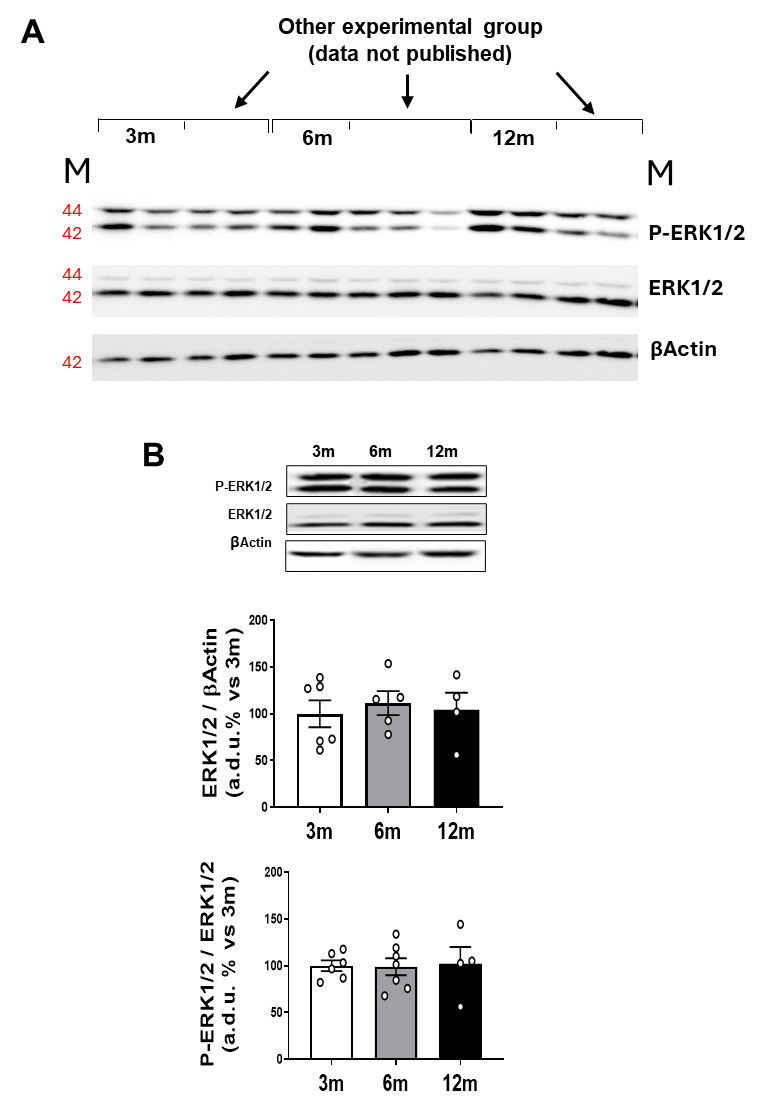


**Supplementary Fig. 15 ERK1/2 protein and its activation (P-ERK1/2 / ERK1/2). A.** Full uncropped blots image of P-ERK1/2, ERK1/2 and βActin. **B.** Densitometric evaluation of ERK1/2 protein and its activation (P-ERK1/2 / ERK1/2) from whole brainstem extract of 3-, 6- and 12-month-old mice. The upper panels display representative Western blots. Results are expressed as arbitrary densitometric units (a.d.u.) relative to the 3-month-old group. Differences across ages were analyzed using one-way ANOVA. No significant differences were found. Data are presented as means ± SEM of 4-6 animals per group. M, molecular weight marker.


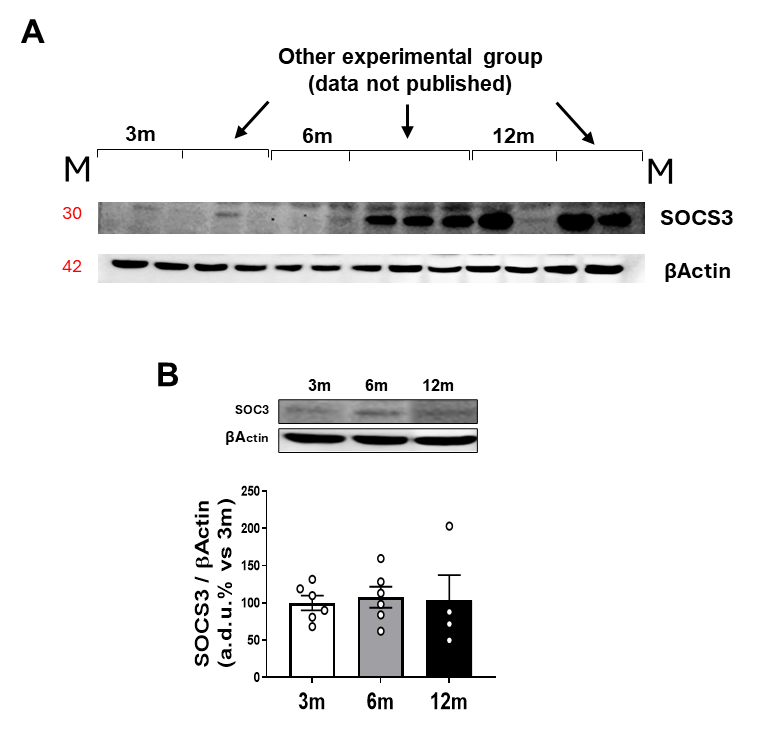


**Supplementary Fig. 16 SOCS3 protein expression. A.** Full uncropped blots image of SOCS3 and βActin. **B.** Densitometric evaluation of SOCS3 protein expression from whole brainstem extract of 3-, 6- and 12-month-old mice. The upper panels display representative Western blots. Results are expressed as arbitrary densitometric units (a.d.u.) relative to the 3-month-old group. Differences across ages were analyzed using one-way ANOVA. No significant differences were found. Data are presented as means ± SEM of 4-6 animals per group. M, molecular weight marker.


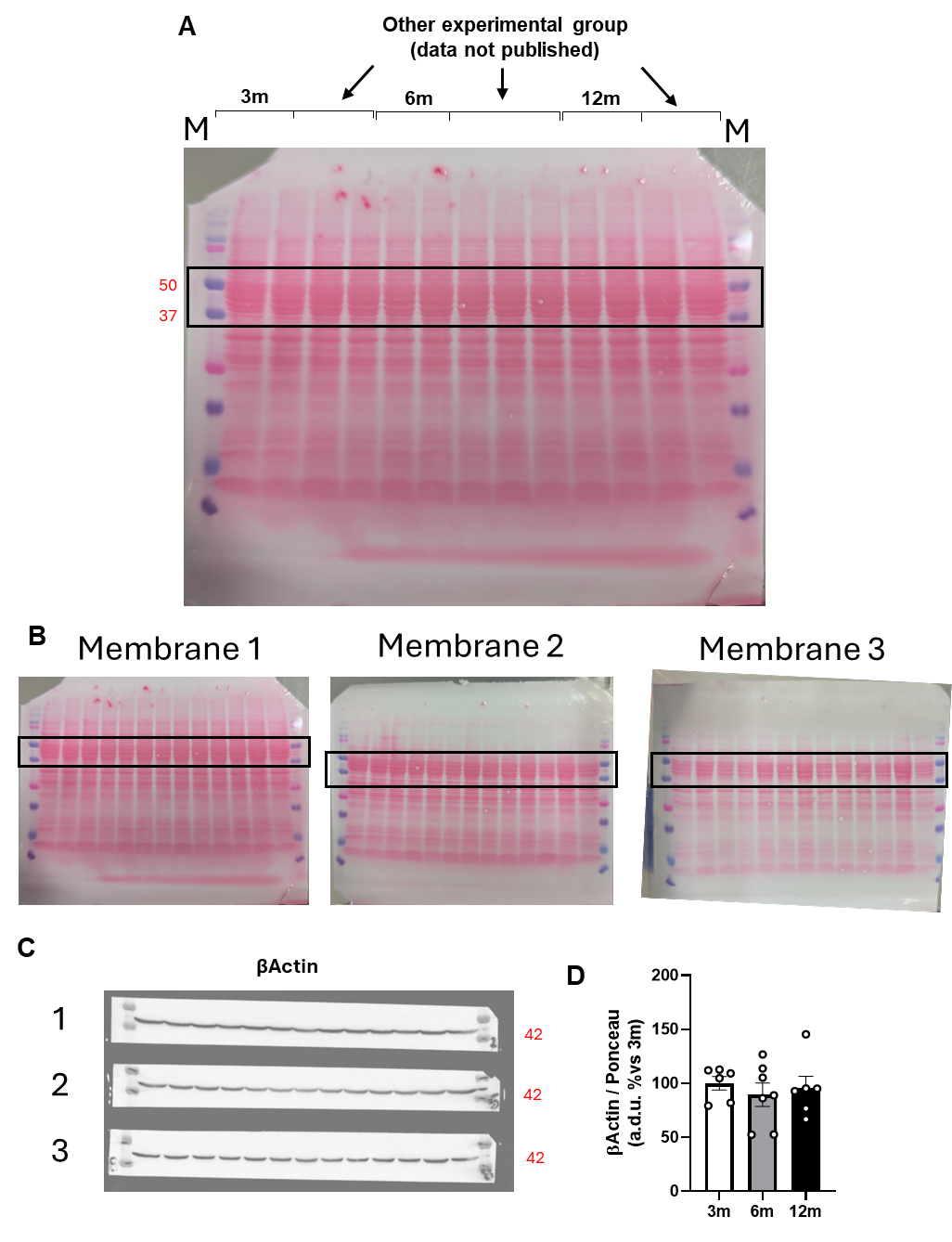


**Supplementary Fig. 17 βActin protein expression**. **A.** Schematic representation of the positioning of experimental groups in the gels. **B.** Membrane 1, 2, and 3 stained with Ponceau Red and membrane fragment used to quantify the intensity of red as an indicator of total protein content of each sample. **C.** Full uncropped blots images. Membranes were sectioned according to the molecular weight of the proteins of interest. Membrane fragments from different gels were simultaneously incubated using the same primary β-actin and secondary antibody. **D.** Densitometric quantification of β-actin protein after correction by total protein amount detected by Ponceau red staining. Results are expressed as arbitrary densitometric units (a.d.u.) as percentage vs. 3-month-old group. Differences in response across ages were analyzed using one-way ANOVA. No significant differences were found in β-actin protein expression across ages. M, molecular weight marker.


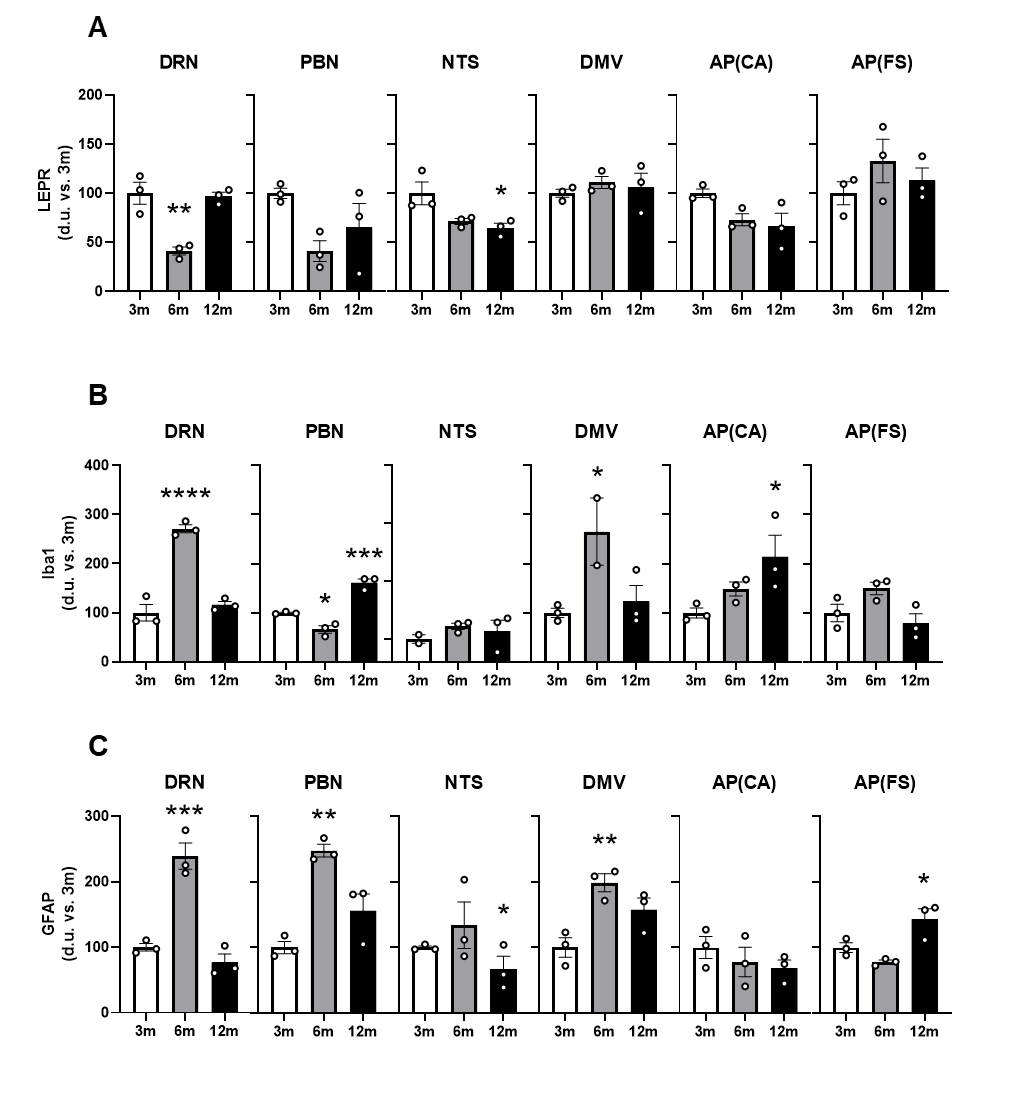


**Supplementary Fig. 18 Quantitative analysis of LEPR (A), Iba1 (B) and GFAP (C) immunoreactivity.** DAB-immunostained slices within each ROI were quantified using ImageJ, expressed as the percentage of positive area relative to 3-month-old animals. Differences across ages in each individual ROI were analyzed using one-way ANOVA followed by Dunnett’s post hoc test. *p<0.05, **p<0.01, ***p<0.001, ****p<0.0001 compared with 3-month-old mice. Data are presented as means ± SEM of 2-3 animals per group.

**Supplementary Table 1 F-statistics and p-values from one-way ANOVA corresponding to the data presented in Table 1.**

| **Results Table 1** | **F (DFn, DFd)** | **P-value** |
| --- | --- | --- |
| **Body Weight (g)** | (2, 36) = 15.33 | <0.0001 |
| **Long (mm)** | (2, 33) = 11.82 | 0.0001 |
| **sWAT (g)** | (2, 11) = 5.068 | 0.0275 |
| **eWAT (g)** | (2, 9) = 7.2700 | 0.0132 |
| **pWAT (g)** | (2, 11) = 7.420 | 0.0091 |
| **Visceral adiposity (%)** | (2, 10) = 8.183 | 0.0078 |
| **Serum leptin levels** | (2, 13) = 15.23 | 0.0004 |
| **Fasting Blood glucose (mg/dl)** | (2, 65) = 9.884 | 0.0002 |
| **Serum insulin levels** | (2, 9) = 4.6210 | 0.0416 |
| **HOMA-IR** | (2, 24) = 15.23 | <0.0001 |

F (DFn, DFd) and P value from F. Dfn is the degree of freedom for the numerator and DFd is the degree of freedom for the denominator. Only statistically significant values are shown (p < 0.05).

**Supplementary Table 2 Statistical values obtained from the results of each figure**

| **Figures** | **F (DFn, DFd); t, df** | **P value** |
| --- | --- | --- |
| **Fig.2 B P-IR / IR 3m** | t=5.579; df=8 | 0.0005 |
| **Fig.2 B P-IR / IR 6m** | t=2.563; df=8 | 0.0335 |
| **Fig.2 B P-IR / IR 12m** | t=4.261; df=10 | 0.0017 |
| **Fig. 2 C** | F (2, 11) = 10.49 | 0.0028 |
| **Fig.2 D P-AKT / AKT 3m** | t=2,357; df=8 | 0.0461 |
| **Fig. 2 F P-AMPK / AMPK 3m** | t=2.346; df=8 | 0.0470 |
| **Fig. 2 F P-AMPK / AMPK 6m** | t=3.699; df=8 | 0.0061 |
| **Fig. 2 F P-AMPK / AMPK 12m** | t=5.322; df=10 | 0.0003 |
| **Fig. 2 G** | F (2, 13) = 13.50 | 0.0007 |
| **Fig.2 H P-STAT3 / STAT3 3m** | t=4.923; df=7 | 0.0017 |
| **Fig.2 H P-STAT3 / STAT3 6m** | t=3.999; df=7 | 0.0052 |
| **Fig. 2 I** | F (2, 11) = 25.31 | <0.0001 |
| **Fig.2 J P-AKT / AKT 3m** | t=5.853; df=6 | 0.0011 |
| **Fig. 2 K** | F (2, 12) = 12.17 | 0.0013 |
| **Fig. 2 L P-AMPK / AMPK 3m** | t=3.871; df=8 | 0.0047 |
| **Fig. 2 L P-AMPK / AMPK 6m** | t=3.537; df=10 | 0.0054 |
| **Fig. 2 M** | F (2, 14) = 5.499 | 0.0173 |
| **Fig. 3 B** | F (2, 10) = 52.38 | <0.0001 |
| **Fig. 3 C P-IR / IR** | F (2, 13) = 8.226 | 0.0049 |
| **Fig. 3 D STAT3 / βActin** | F (2, 14) = 7.046 | 0.0076 |
| **Fig. 3 E IRS1 / βActin** | F (2, 12) = 10.23 | 0.0026 |
| **Fig. 3 F AKT / βActin** | F (2. 13) = 3.187 | 0.0748 |
| **Fig. 3 F P-AKT / AKT** | F (2, 13) = 8.367 | 0.0046 |
| **Fig. 3 G P-GSK3 / GSK3** | F (2, 13) = 4.845 | 0.0268 |
| **Fig. 3 H mTOR / βActin** | F (2. 15) = 8.903 | 0.0028 |
| **Fig. 3 H P-mTOR / mTOR** | F (2, 12) = 5.698 | 0.0182 |
| **Fig. 3 I p70 / βActin** | F (2, 14) = 4.493 | 0.0311 |
| **Fig. 3 J P-PKCε /PKCε** | F (2, 14) = 8.312 | 0.0042 |
| **Fig. 3 K AMPK / βActin** | F (2, 14) = 4.332 | 0.0343 |
| **Fig. 3 L PTEN / βActin** | F (2, 14) = 4.378 | 0.0334 |
| **Fig. 4 B** | F (2, 9) = 108.8 | <0.0001 |
| **Fig. 5 A** | Interaction F (4, 29) = 1.149 | 0.3534 |
|  | Inflammatory markers F (2, 29) = 3.746 | 0.0357 |
|  | Age F (2, 29) = 10.61 | 0.0003 |
| **Fig. 5 B Relative phosphorilated protein expression** | Interaction F (2, 28) = 0.1883 | 0.8294 |
|  | Inflammatory markers F (1, 28) = 0.3698 | 0.5480 |
|  | Age F (2, 28) = 19.53 | <0.0001 |
| **Fig. 6 B** | F (2, 10) = 24.81 | 0.0001 |
| **Fig. 7 B** | F (2, 10) = 6.318 | 0.0168 |

Only statistically significant values are shown (p < 0.05). Student's t-tests analyses: t-statistics (t), degree of freedom (df) and p values. ANOVA : F (DFn, DFd) and P value from F. Dfn is the degree of freedom for the numerator and DFd is the degree of freedom for the denominator.

**Supplementary Table 3 Statistical values obtained by one-way ANOVA test analyses from the results of each IHC figure.**

| **IHC Figures** | | **F (DFn, DFd)** | **P value** |
| --- | --- | --- | --- |
| **LEPR** | DRN | F (2, 6) = 20,10 | P=0,0022 |
|  | NTS | F (2, 6) = 6,295 | P=0,0336 |
| **Iba1** | DRN | F (2, 6) = 67,00 | P<0,0001 |
|  | PBN | F (2, 6) = 59,56 | P=0,0001 |
| **GFAP** | DRN | F (2, 6) = 38,39 | P=0,0004 |
|  | PBN | F (2, 6) = 19,90 | P=0,0022 |
|  | DMV | F (2, 6) = 10,09 | P=0,0120 |
|  | AP (FS) | F (2, 6) = 10,47 | P=0,0110 |

Only statistically significant values are shown (p < 0.05). F (DFn, DFd) and P value from F; Dfn is the degree of freedom for the numerator of the F ratio, and DFd is for the denominator.
